# Resistance Mutation Profiles Associated with Current Treatments for Epidermal Growth Factor Receptor-Mutated Non-Small-Cell Lung Cancer in the United States: A Systematic Literature Review

**DOI:** 10.3390/curroncol32040191

**Published:** 2025-03-25

**Authors:** Pratyusha Vadagam, Dexter Waters, Anil Bhagat, Yuting Kuang, Jennifer Uyei, Julie Vanderpoel

**Affiliations:** 1 Johnson & Johnson, 800 Ridgeview Dr, Horsham, PA 19044, USA; 2 IQVIA Inc., 1850 Gateway Drive, San Mateo, CA 94404, USA

**Keywords:** non-small-cell lung cancer, EGFR, resistance mechanism, tyrosine kinase inhibitor, resistance mutation profile, clinical outcomes

## Abstract

Treatment resistance due to gene alterations remains a challenge for patients with EGFR-mutated advanced or metastatic non-small-cell lung cancer (a/mNSCLC). A systematic literature review (SLR) was conducted to describe resistance mutation profiles and their impact on clinical outcomes in adults with a/mNSCLC in the United States (US). A comprehensive search of MEDLINE and Embase (2018–August 2022) identified 2986 records. Among 45 included studies, osimertinib was the most commonly reported treatment (osimertinib alone: 15 studies; as one of the treatment options: 18 studies), followed by other tyrosine kinase inhibitors (TKIs; 5 studies) and non-TKIs (1 study). For first-line (1L) and second-line (2L) osimertinib, the most frequent EGFR-dependent resistance mechanisms were T790M loss (1L: 15.4%; 2L: 20.5–49%) and C797X mutation (1L: 2.9–12.5%; 2L: 1.4–22%). EGFR-independent mechanisms included MET amplification (1L: 0.6–66%; 2L: 7.2–19%), TP53 mutation (1L: 29.2–33.3%), and CCNE1 amplification (1L: 7.9%; 2L: 10.3%). For patients receiving osimertinib, EGFR T790M mutation loss, EGFR/MET/HER2 amplification, RET fusion, and PIK3CA mutation were associated with worse progression-free survival. Resistance mechanisms resulting from current NSCLC treatments in the US are complex, underscoring the need to address such heterogeneous resistance profiles and improve outcomes for patients with EGFR-mutated a/mNSCLC.

## 1. Introduction

Lung cancer is the most common cause of cancer death in the United States (US) with an estimated 238,340 new cases and 127,070 deaths in 2023 [1]. Non-small-cell lung cancer (NSCLC) is the most common sub-type of lung cancer, accounting for approximately 80–85% of all cases [2]. Widespread screening has the potential to diagnose earlier-stage cancers; however, more than half of newly diagnosed patients with lung cancer have advanced or metastatic disease [3]. One of the most common oncogenic drivers in NSCLC is mutation in the epidermal growth factor receptor (EGFR). In a cohort of patients with recurrent or metastatic lung adenocarcinoma, EGFR alterations occurred in up to 28% of patients, although its prevalence could be largely impacted by factors such as age, sex, race, and smoking status [4].

Molecular testing in NSCLC is now considered standard of care, and genome-sequencing approaches have led to the discovery of a growing number of oncogenic mutations [5]. The majority of the oncogenic drivers in NSCLC represent somatic events that result in the hyperactivation of a kinase and oncogene addiction in the tumor cells. Therefore, targeted therapies have a significantly larger therapeutic window than traditional cytotoxic chemotherapy [6]. Studies have demonstrated clinical efficacy with second- and third-generation tyrosine kinase inhibitors (TKIs) [7,8,9]. Most recently, osimertinib has been found to have overall survival (OS) benefit over first-generation EGFR targeting TKIs and is associated with a favorable tolerability profile, positioning it as a standard first-line treatment option for common sensitizing EGFR mutations including exon 19 deletion and exon 21 L858R (L858R) [10].

Considering the development of targeted therapies, the National Comprehensive Cancer Network (NCCN) recommends that all patients with metastatic non-squamous NSCLC undergo biomarker testing for oncogenic drivers including ALK rearrangements, BRAF mutations, EGFR mutations, HER2 mutations, and ROS1 rearrangement. However, mutations can emerge during treatment and trigger acquired resistance to targeted therapy, but such acquired mutations have not been commonly assessed among patients with disease progression and remain to be further characterized.

Several mechanisms capable of triggering acquired resistance have been described in patients with EGFR-mutated advanced NSCLC, such as EGFR T790M mutation, EGFR C797S mutations, MET amplification, and small-cell transformation. These mechanisms, which may be EGFR-dependent or EGFR-independent, are often associated with poor prognoses, including shorter progression-free survival (PFS). In EGFR-dependent resistance mechanisms, tumor cell proliferation depends largely on EGFR signaling. On the other hand, EGFR-independent resistance mechanisms are characterized by the predominance of other parallel pathways that bypass EGFR signaling.

Despite the emerging targeted therapies in treating EGFR-mutated NSCLC, treatment resistance remains a challenge for a subset of patients due to several factors, including reduced sensitivity to TKIs and the presence of concurrent genetic alterations, such as MET amplifications or modifiers of response to TKIs [10]. Post-osimertinib, pharmacological options become limited, with chemotherapy remaining the main therapeutic strategy [11]. In view of this, a comprehensive understanding of the current landscape of resistance mechanisms for EGFR-mutated NSCLC is needed to allow for the identification of unmet medical needs. A systematic literature review (SLR) was conducted to describe the resistance mutation profiles and impact of resistance mutations on clinical outcomes in adults with locally advanced or metastatic NSCLC (a/mNSCLC) with EGFR mutation. Considering potential geographical differences in population characteristics, treatment landscape, and healthcare settings, this review focused on studies involving patients in the US.

## 2. Materials and Methods

An SLR was conducted following the methods outlined in the Cochrane Handbook for Systematic Reviews of Interventions [12] and the Preferred Reporting Items for Systematic Reviews and Meta-Analyses (PRISMA) statement [13]. A detailed protocol was developed prior to conducting the review and was registered prospectively in PROSPERO (registration ID CRD 42022355822).

A comprehensive search was performed in MEDLINE^®^ Epub Ahead of Print, In-Process & Other Non-Indexed Citations, Medline^®^ Daily, Medline and Versions^®^, and EMBASE by using the OvidSP^®^ platform to identify relevant studies. The databases were searched from 2018 to 3 August 2022 and limited to English language publications (complete search strategies are presented in Supplementary Table S1). Since osimertinib was approved by the Food and Drug Administration in 2018 for the first-line treatment of metastatic NSCLC with EGFR mutation, only evidence from 2018 onwards was included to reflect the resistance mutation profiles associated with the more recent treatment landscape. In addition to the bibliographic databases, websites of the following selected conferences for the most recent meetings were searched: American Society of Clinical Oncology (ASCO) Annual Meeting, European Society for Medical Oncology (ESMO) Congress, World Conference on Lung Cancer (WCLC), International Society for Pharmacoeconomics and Outcomes Research (ISPOR) conference, and Academy of Managed Care Pharmacy (AMCP) Annual Meeting. The bibliographies of recently published reviews on the related topic area were also reviewed to help mitigate the risk of publication bias and to identify as much relevant evidence as possible [12].

Studies were eligible for inclusion if they included adults (18 years or older) with EGFR-mutated a/mNSCLC. There were no restrictions for interventions or comparators. Clinical trials, observational studies, and genome-sequencing studies that were published between 2018 and 3 August 2022 were included. For geography, only studies conducted in the US or those including at least a proportion of patients from the US were included. Case reports and review articles were excluded, and so were studies that did not include patients in the US and studies that did not include human subjects.

Two reviewers working independently reviewed the title and abstract of all unique studies identified by the search. Records were excluded if they did not meet pre-defined inclusion criteria as assessed by both reviewers; records were retained for full-text review if assessed as definitely or possibly relevant by both reviewers. Further, two reviewers working independently assessed each full-text publication to determine eligibility, and reasons for exclusion were documented. At any stage, records for which there was uncertainty about inclusion/exclusion or there was a discrepancy between the two reviewers, a third reviewer adjudicated a decision to include or exclude. The process of search and screening was summarized in a PRISMA flow diagram. Data extraction was conducted by two independent reviewers and discrepancies were checked against the source document by a third reviewer. Publications reporting results for the same study were grouped per study.

Key data on methodological characteristics, selection criteria, study population/patient characteristics, and results were extracted from the included studies. Number and proportion of patients with different mechanisms of resistance mutation and co-mutations were extracted. Key outcomes of interest were resistance mutation profile (mechanisms of acquired resistance, especially to EGFR tyrosine kinase inhibitors [EGFR TKIs]) and the impact of resistance mutation on clinical outcomes (OS, PFS, overall response rate [ORR], complete response [CR], partial response [PR], duration of response, time to next treatment, and time to treatment discontinuation [TTD]). Acquired resistance mechanisms and alterations were recorded as defined by the study authors and not limited to paired samples of the same testing modality.

Risk of bias for the included non-randomized and randomized studies was assessed according to the Newcastle-Ottawa scale (NOS) [14] and Cochrane Risk of Bias Tool (RoB2) [15], respectively.

## 3. Results

### 3.1. Study Selection

**Figure 1:**
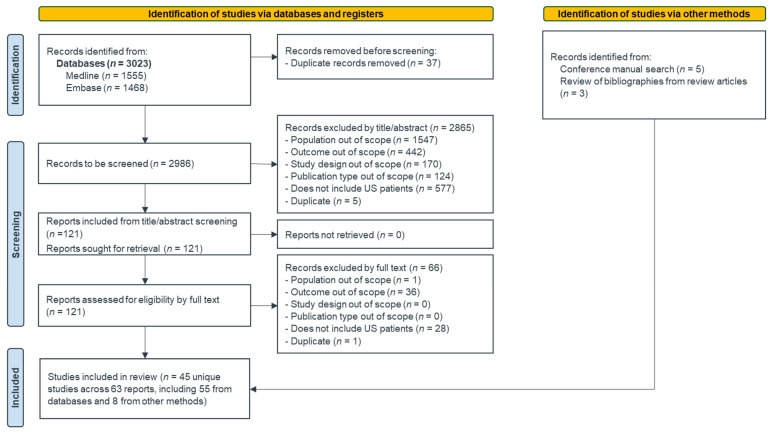
PRISMA flow diagram.

The search and screening results are illustrated in the PRISMA flow diagram in Figure 1. A total of 3023 publications were identified through database searches. After de-duplication, 2986 unique records were retained for title and abstract review. A total of 121 records were retained for full-text review, and 8 additional records meeting the eligibility criteria were identified from manual searches of conferences and bibliographies of review articles. After full-text review, 63 eligible publications reporting data on 45 unique studies were included for data extraction.

### 3.2. Study and Patient Characteristics

Study and patient characteristics are summarized in Table 1, with additional information in Supplementary Table S2. The majority of the included studies were observational studies (77.8%, *n* = 35) [16,17,18,19,20,21,22,23,24,25,26,27,28,29,30,31,32,33,34,35,36,37,38,39,40,41,42,43,44,45,46,47,48,49,50], and the rest were single-arm trials (8.9%, *n* = 4) [51,52,53,54], non-randomized clinical trials (8.9%, *n* = 4) [55,56,57,58], or randomized clinical trials (RCTs) (4.4%, *n* = 2) [8,59]. Twenty-three (51.1%) studies were published as journal articles, and twenty-two (48.9%) were published as conference abstracts. Twenty-seven studies (60%) included US-only populations, and eighteen (40%) studies included a mix of US populations and other geographies/countries. Fifteen studies reported on osimertinib alone, and eighteen included osimertinib as one treatment option among patients treated with TKIs or non-TKI treatment. Six studies reported on treatments other than osimertinib, of which five reported on TKIs including poziotinib, rociletinib, aumolertinib, capmatinib, and erlotinib and one reported on non-TKIs. Treatment type was not specified in six studies.

The majority of studies (64%) reported on the line of therapy (LoT). A total of 6 studies investigated first-line therapy alone, 18 studies investigated first-line therapy and beyond, 1 study reported second-line therapy alone and 4 studies reported second-line therapy and beyond. The line of therapy was not specified in 16 studies. The proportion of males ranged from 25% to 85.5% among the included studies. Where reported, the median age ranged from 59 years to 69.5 years. The study period varied across studies and ranged from 2005 to 2022, with a few studies ongoing beyond 2022. The sample size ranged from 9 to 16,715.

### 3.3. Quality Assessment of Included Studies

The two RCTs were deemed as having some concern for risk of bias due to missing outcome data in the individual studies (see Supplementary Figure S1). Of the 29 studies that did not report clinical outcomes, out of a maximum of 4 stars, the total NOS score was 3 for most studies, suggesting a low risk of bias. A total of 6 studies had 1 star (high risk of bias), 7 studies had 2 stars (medium risk of bias), 13 studies had 3 stars (low risk of bias), and 3 studies had 4 stars (low risk of bias).

Of the 14 observational studies that reported clinical outcomes, out of a maximum of 6 stars, the total NOS score was 2 or 3 stars for most studies, suggesting a medium risk of bias. Two studies had 1 star (high risk of bias), five studies had 2 stars (high risk of bias), five studies had 3 stars (medium risk of bias), and one study each had 4 or 5 stars (low risk of bias) (see Supplementary Table S3).

### 3.4. Outcomes

#### 3.4.1. Acquired Resistance Mutation Profile by Line of Therapy

**First-Line osimertinib**

**Figure 2:**
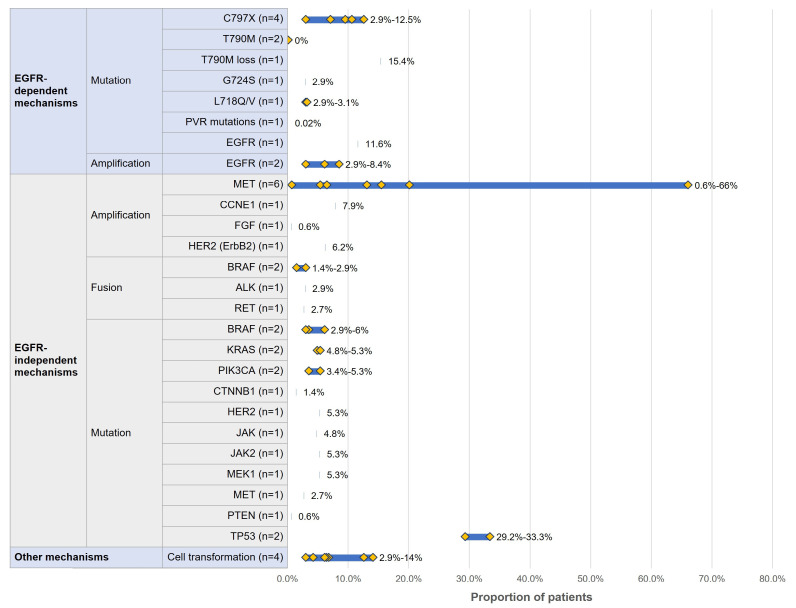
Resistance mechanisms for patients who received first-line osimertinib. n = number of studies that reported each resistance mutation; the blue bars show the range for proportion of patients with a detected resistance mutation across studies; the yellow diamonds indicate the proportion of patients with a specific resistance mutation in each study for cases with multiple studies; C797X: broad category inclusive of C797S and C797G mutations.

Eight studies [8,18,37,39,40,42,54,58] reported the proportion of acquired EGFR-dependent and EGFR-independent resistance mechanisms in patients who received first-line osimertinib. As shown in Figure 2, the resistance mutation profile of patients who received first-line osimertinib was heterogenous and complex. Among the EGFR-dependent mechanisms, T790M loss (15.4%) was the most frequently occurring alteration [18], followed by C797X mutation (2.9–12.5%) [8,37,39,54] and acquired EGFR mutations (11.6%; type unspecified) [18]. Notably, T790M loss was reported as a common molecular alteration at the time of progression to first-line osimertinib [18]. Other mechanisms that occurred less frequently included EGFR amplification (2.9–8.4%) [18,37], L718Q/V mutation (2.9–3.1%) [37], acquired G724S (2.9%) [37], and pocket volume reducing mutation (0.02%) [40]. When osimertinib was given as first-line treatment, no evidence for acquired T790M mutation was detected at resistance (0%) [8,54].

Among the EGFR-independent mechanisms, the most commonly occurring one was MET amplification (0.6–66%) [8,18,37,39,54,58], followed by TP53 mutation (29.2–33.3%) [18,58], CCNE1 amplification (7.9%) [39], HER2 amplification (6.2%) [18], BRAF mutation (2.9–6.0%) [18,37], HER2 exon 20 insertion (5.3%) [54], JAK2 V617F mutation (5.3%) [54], MEK1 mutation (5.3%) [54], KRAS mutation (4.8–5.3%) [18,54], and PIK3CA mutation (3.4–5.3%) [18,54].

Overall, the proportion of patients who experienced co-occurring acquired resistance mechanisms varied from 2.0% to 40.8% (Supplementary Table S4). The proportion of patients who experienced other resistance mechanisms, including cell transformation, were reported to range from 2.9% to 14%, where histologic transformation could potentially be another predominant mechanism of resistance with frequency comparable to other common EGFR-dependent mechanisms. Additional details and information on these mechanisms can be found in Supplementary Table S4.

**Second-Line osimertinib**

**Figure 3:**
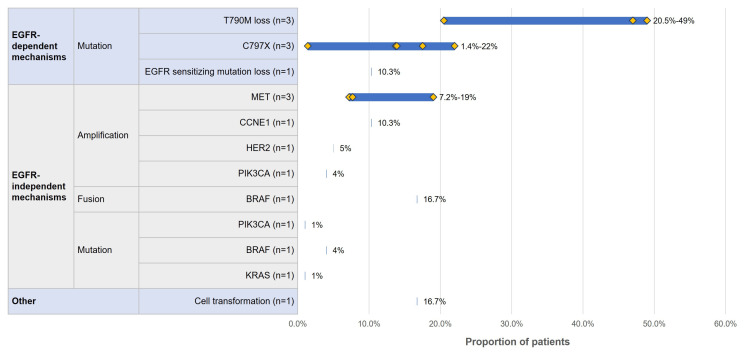
Resistance mechanisms for patients who received second-line osimertinib. n = number of studies that reported each resistance mutation; the blue bars show the range for proportion of patients with a detected resistance mutation across studies; the yellow diamonds indicate the proportion of patients with a specific resistance mutation in each study for cases with multiple studies; C797X: broad category inclusive of C797S and C797G mutations.

Five studies [24,33,39,58,59] reported the proportion of acquired EGFR-dependent and EGFR-independent resistance mechanisms in patients who received second-line osimertinib. The proportion and range of patients who presented with individual resistance mechanisms are presented in Figure 3, with additional details and co-occurring mutations presented in Supplementary Table S5.

Similar to the resistance mutation profile for patients who received first-line osimertinib, T790M loss (20.5–49%) [24,33,59] was the most frequently occurring EGFR-dependent mechanism, followed by C797X mutation (1.4–22%) [33,39,59] and EGFR sensitizing mutation loss (10.3%) [24]. In this setting, among patients who had T790M-positive disease following first-line therapy, the status of T790M mutation was evaluated at the time of resistance to second-line osimertinib. While two studies reported the observation of T790M loss without further characterization [24,59], in one study, the loss of T790M mutation was reported to mediate the acquired resistance to second-line osimertinib and associate with competing mechanisms [33]. Interestingly, acquired C797S mutation was only observed in patients who maintained T790M in one study, where the results were stratified by T790M status [33].

The most commonly occurring EGFR-independent mechanisms among patients who received second-line osimertinib were similar to those reported in patients who received first-line osimertinib. The most commonly occurring mechanism was MET amplification (7.2–19%) [33,39,59], followed by CCNE1 amplification (10.3%) [39]. The proportion of patients with other mechanisms, including cell transformation, was 16.7% [58] (see Supplementary Table S5).

**Resistance mutation profile for patients who received other lines of osimertinib**

Several studies reported resistance mechanisms for other lines of osimertinib. Six studies [17,27,34,36,42,58] reported the proportion of acquired EGFR-dependent and EGFR-independent resistance mechanisms in patients who received first-line osimertinib and beyond—defined as osimertinib as first-line treatment or after prior treatment—and five studies [29,33,42,50,55] reported the proportions in patients who received second-line osimertinib and beyond (Supplementary Tables S6 and S7).

The most frequently occurring EGFR-dependent mechanisms were T790M loss (first-line and beyond: 23.9–50% [17,27,34,42]; second-line and beyond: 30.9–68.3% [33,42,50]) and C797X (first-line and beyond: 11.1–32.0% [17,27,34,36]; second-line and beyond: 11–22.0% [29,33,50]). Notably, the reporting of T790M loss varied across studies. The loss of T790M mutation was reported as a resistance mechanism in two studies [34,42], as an alteration observed at osimertinib progression in two studies [17,50], and as a mediator of acquired resistance in two studies, where the mutation profile was further stratified by T790M status [27,33]. When stratified, the mutation profile for T790M-loss cases were reported to be more diverse and involving EGFR-independent mechanisms, while for T790M-preserved cases, EGFR tertiary mutations were more commonly observed [27,33]. EGFR C797X mutation [27,33] and L792H mutation [27] were considered previously defined osimertinib-resistant EGFR mutations by the study authors and were observed exclusively in T790M-preserved cases.

MET amplification (10.0–23.0%) [17,27,34,36] was reported to be the most frequently occurring EGFR-independent mechanism in patients who received first-line osimertinib and beyond. When stratified by T790M status at resistance, MET amplification was observed in 26.3% of T790M-preserved cases and 4.8% of T790M-loss cases [27]. Among patients who received osimertinib in the second-line therapy and beyond setting, PI3K-AKT-mTOR signaling activating mutation (9.8–12.6%) [33,50] was most frequently occurring, followed by MET amplification (5.5–9.8%) [29,33,50]. When further stratified by T790M status at resistance, PIK3CA mutation was observed in 15.4% of T790M-preserved cases and 7.1% of T790M-loss cases, while MET amplification was observed in 0% of T790M-preserved cases and 14.3% of T790M-loss cases in this setting [33]. Additionally, eight studies that included patients who received an unspecified line of osimertinib reported trends similar to those reported in patients who received first-line osimertinib.

**Resistance mutation profile for patients who received other treatments**

Several studies reported on the proportion of EGFR-dependent and EGFR-independent mechanisms for different combinations of treatments. Eighteen studies [8,16,21,30,31,32,35,36,40,41,43,48,57,58,59] included patients who received TKIs or non-TKIs (including chemotherapy and immunotherapy) where osimertinib was one of the treatment options, six studies [23,51,52,53,55,56] included patients who received other TKIs or non-TKIs, and another six studies [19,20,26,28,38,45] included patients for whom the treatment type was not specified (Supplementary Tables S8–S10).

The most frequently occurring EGFR-dependent mechanisms reported in these studies differed from what was reported for patients who received first-line or second-line osimertinib. For patients who received TKIs or non-TKIs where osimertinib was one of the treatment options, C797X mutation (6.9–79.3%) [21,57] and T790M mutation (32–72%) [32,35] were the most frequently occurring EGFR-dependent mechanisms.

Beyond treatments involving osimertinib, other TKI and non-TKI therapies have also been associated with various resistance mechanisms. For patients who received other TKIs and non-TKIs, T790M mutation (13–90.8%) [35,51] was the most frequently occurring EGFR-dependent mechanism followed by C797X (4.6–16.7%) [23,52]. One study [23] also reported that EGFR alteration was observed in 36% of patients, but the type of alteration was not specified. Similar to patients who received other TKIs and non-TKIs, T790M mutation (0.9–76%) [35,38] was the most frequently occurring EGFR-dependent mechanism in patients who received unspecified treatments. One study reported EGFR mutation in 4.0–19.1% [19] of patients who received unspecified treatments, but the specific type of mutation was not reported.

EGFR-independent mechanisms varied across treatment categories. For patients who received TKIs or non-TKIs where osimertinib was one of the treatment options, the most frequently occurring EGFR-independent mechanisms were PIK3CA alteration (8.9–44.4%) [30,31], RB1 alteration (33.3%) [31], FGFR3 fusion (0.7–33.3%) [30,43], and ALK fusion (0.2–25%) [30,43], while TP53 alteration (45%) [23] and RET fusion (29–71%) [47] were the most frequently occurring mechanisms in patients who received other TKIs/non-TKIs or unspecified treatment, respectively.

Specifically, five studies that exclusively evaluated patients receiving treatments other than osimertinib reported results and revealed complex resistance mutation profiles. Helman et al. [23] observed multiple resistance mechanisms among patients treated with rociletinib in the TIGER-X and TIGER-2 studies, including C797S mutations (4.6%), KRAS/NRAS/HRAS mutations (14%), an NTRK1 fusion (2%), and MET amplification (7.6%). The APOLLO study [52] identified C797S mutations in 16.7% of patients treated with aumolertinib. A phase 2 study [51] of poziotinib reported that 47.8% of patients had EGFR-independent resistance mechanisms and 17.4% had EGFR-dependent resistance mechanisms, including acquired EGFR gatekeeper mutations (13%). Interestingly, C797S mutation was not observed as a resistance mechanism in this study. A phase 1/2 study [53] of capmatinib in combination with erlotinib in patients with MET-positive NSCLC observed acquired T790M mutations in 8.3% of patients. Among patients who had progressed on osimertinib and had been treated with a combination of amivantamab and lazertinib in the CHRYSALIS study [55], 22.2% had non-EGFR/MET mechanisms of osimertinib resistance, and none of these patients responded to treatment. Additionally, 40% were observed to have unknown mechanisms of resistance.

#### 3.4.2. Impact of Acquired Resistance on Clinical Outcomes

Fourteen studies reported clinical outcomes for patients with different types of treatment and acquired resistance mechanisms: response (nine studies) [16,20,23,40,43,46,53,55,56], PFS (eight studies) [18,20,23,26,33,40,47,55], TTD (two studies) [32,33], and OS (one study) [32]. Four studies evaluated the impact of acquired resistance mechanisms on clinical outcomes statistically (Supplementary Table S11). Among these four studies, two reported on osimertinib [18,33], and two reported on other EGFR TKIs or chemotherapy [20,40].

In one study of patients who received osimertinib for acquired EGFR T790M resistance mutation to a prior EGFR TKI, the loss of T790M compared with maintained T790M was associated with worse PFS (median of 4.2 vs. 9.5 months, *p* = 0.001) and shorter TTD (6.1 months vs. 15.2 months, *p* = 0.01) [33]. In this study, loss of the T790M mutation was associated with early resistance to osimertinib. In another study that evaluated first-line osimertinib, EGFR amplification, MET amplification, RET fusion, HER2 amplification, and PIK3CA mutation were associated with worse PFS (the median PFS ranged between 4.63 and 15.1 months across subgroups of patients with or without these resistance alterations) [18]. The presence of TP53 mutations and the loss of EGFR T790M were the most common molecular alterations at the time of progression. While TP53 mutation did not have an impact on PFS in the overall population, among patients with CNS metastases, those with TP53 mutations at progression had a worse PFS compared with those without TP53 mutations (median of 11.0 vs. 24.9 months) [33]. Interestingly, longer PFS was observed in patients with EGFR T790M loss (median of 28.1 months vs. 13.2 months) [33], but no further interpretation was provided by the authors.

For other treatments, the presence of acquired EGFR T790M mutation was associated with improved PFS on first-line TKI treatment or chemotherapy. Based on these findings, the authors suggested that tumors expressing the T790M resistance mutation have a more indolent progression of disease than their T790M-negative counterparts [20]. Additionally, no associations were observed between acquired EGFR T790M mutation and TTD or OS on first-line EGFR TKI treatment [40].

## 4. Discussion

The studies identified in our SLR show that mechanisms of resistance to current NSCLC treatments, primarily osimertinib, in US populations are heterogenous and complex and pose a challenge for treating patients with EGFR-mutated a/mNSCLC. Various mechanisms of resistance were reported in the studies, yet across different lines of osimertinib, T790M loss or C797X mutation was consistently the most frequently reported EGFR-dependent mechanism. Specifically, across studies that reported on resistance to first-line or second-line osimertinib, up to 49% and 22% of patients had T790M loss and C797X, respectively. These estimates are similar to the estimates previously reported by Kobayashi et al. [78]. In a pooled analysis, Kobayashi et al. reported that the incidence of T790M loss after osimertinib treatment in patients with histologically confirmed NSCLC was 58.4% and the incidence of C797S mutation in patients who developed acquired resistance to osimertinib was 21.58%.

Two studies included in the review reported inconsistent results in terms of the clinical impact of T790M loss. Oxnard et al. [33] reported that loss of EGFR T790M mutation was associated with worse PFS and early resistance on second-line osimertinib or beyond. In contrast, Cardona et al. [18] reported that longer PFS was observed in patients with EGFR T790M loss on first-line osimertinib. It is important to note that there are likely biological differences when addressing the T790M mutation in first-line therapy vs. the T790M mutation that develops after using another EGFR TKI. The characterization for T790M loss varied across studies (as resistance mechanism, alteration at progression, or mediator of resistance); nevertheless, the loss of T790M mutation was commonly observed in patients who developed resistance to osimertinib and could be associated with the emergence of different resistance mutation profiles compared with cases with T790M maintained.

In addition, several other EGFR-dependent mechanisms of resistance, including EGFR amplification, mutations in L718Q/V, and G724S, were reported in varying proportions for patients who received first- or second-line osimertinib. Several studies also reported the proportion of patients with EGFR exon 19 deletion, L858R, driver single-nucleotide variant, and exon 20 insertion as part of the mutation profile at progression, without clearly distinguishing EGFR activation mutations and resistance mutations in the report [19,30,53]. Considering that these are typically recognized as EGFR activation mutations, they were not captured as resistance mechanisms.

A variety of EGFR-independent mechanisms were reported across different lines of osimertinib. MET amplification was consistently the most prevalent EGFR-independent mechanism across studies that reported first-line osimertinib only, first-line therapy and beyond, or second-line therapy only, with an observed proportion ranging between 0.6% and 23.0%. This finding is comparable to a pooled analysis published by Kobayashi et al. [78], which reported that 10.65% of patients with NSCLC who progressed on osimertinib had MET amplification. One study in the present review reported a high incidence of 66% for MET amplification among patients receiving first-line osimertinib [58]. While the authors did not provide further interpretation for this high incidence, this observation could be an outlier due to small sample size (*n* = 9) and other potential bias in sample selection. Other EGFR-independent mechanisms that were reported include CCNE1 amplification, HER2 amplification, RET fusion, and mutations in TP53, BRAF, PIK3CA, and KRAS, among several others. In one study, the authors reported that MET amplification, RET fusion, HER2 amplification, and PIK3CA mutation were associated with worse PFS in patients on first-line osimertinib [18].

Overall, the resistance mechanisms developed during osimertinib treatment are complex, involving both EGFR-dependent and EGFR-independent pathways. Common EGFR-dependent mechanisms observed in the reviewed studies include T790M loss, C797X mutation, EGFR amplification, and mutations in L718Q/V and G724S. Common EGFR-independent mechanisms include MET amplification, CCNE1 amplification, HER2 amplification, RET fusion, and mutations in TP53, BRAF, PIK3CA, and KRAS, among others. These mechanisms may involve EGFR alternative bypass pathway activation, downstream pathway activation, oncogene fusion, or cell-cycle gene mutation, contributing to resistance to osimertinib.

In the included studies, these mechanisms were reported as acquired resistance mechanisms or acquired alterations as defined by the study authors, although some variation in the characterization was observed. For example, T790M loss was reported as a resistance mechanism, an alteration at progression, or a mediator of resistance. Variations in the occurrence of these mutations were observed across different lines of osimertinib treatment.

Additionally, multiple co-existing molecular alterations were observed in varying proportions across the included studies, both when osimertinib was administered as first- and second-line therapy. The heterogeneity and co-occurrence of multiple resistance mechanisms constitute a major challenge in developing an efficient treatment strategy to counteract tumor progression. The development of acquired resistance has limited the durability of clinical benefit experienced in patients treated with osimertinib and other TKIs for a/mNSCLC. The available data about resistance mechanisms especially to osimertinib or other TKIs are inconsistent, and published reports have suggested that treatment-naïve patients who progressed on osimertinib or other TKIs should be better characterized. Considering that the effectiveness of osimertinib in patients with a/mNSCLC is limited by the development of a heterogenous array of resistance mutations, there is a need for therapies that can mitigate the development of resistance to first-line osimertinib and/or broadly target the multitude of resistance mechanisms that are acquired after first-line osimertinib.

This SLR highlights several important gaps in the evidence. First, there is paucity of evidence relating to the impact of resistance mechanisms on clinical outcomes for patients with EGFR-mutated NSCLC. Of all the studies included in this SLR, none were specifically designed to measure the impact of resistance mechanisms on clinical outcomes such as ORR, CR, and PR. Second, factors such as race/ethnicity, social determinants of health, smoking status, and presence of brain metastases were infrequently reported. More evidence is needed to understand the impact of resistance mechanisms on clinical outcomes for patients with EGFR-mutated NSCLC, as well as patient, social, and behavioral factors that may be associated with particular resistance mechanisms. Finally, more evidence is needed to understand the occurrence of brain metastases by resistance mechanism, a common complication among patients with NSCLC.

This SLR has a few limitations. First, this SLR focused on studies that include US populations; therefore, the results of this SLR may not be generalizable globally. Second, the included studies are heterogenous in terms of study and patient characteristics, which could have an impact on the consistency of the results. However, when presenting the results, we have indicated data from individual studies to show the overall trend and potential outliers. In addition, there was limited evidence identified that focused exclusively on treatments other than osimertinib, but that is reflective of the current treatment practice, as our SLR included publications between 2018 and 2022.

## 5. Conclusions

The available literature on resistance mechanisms associated with current NSCLC treatments, primarily osimertinib, reveals molecular patient profiles that are heterogenous and complex, which may pose challenges in improving clinical outcomes in these patients in the US. While the evidence is limited, some resistance mutations have been associated with worse PFS, such as MET amplification, RET fusion, HER2 amplification, and PIK3CA mutation in patients on first-line osimertinib. In addition, T790M loss was associated with worse PFS in patients on 2L osimertinib after a prior EGFR TKI. These findings highlight the need to address both EGFR-dependent and EGFR-independent resistance mechanisms to improve outcomes for patients with EGFR-mutated a/mNSCLC.

## Figures and Tables

**Table 1:** Study and patient characteristics.

Author Year; Country	LoT; Treatment	Study Design	Study Period	N	Sex, Male %	Age, Years-Median (Range)
Osimertinib						
Cardona 2022; international [18]	1L; osimertinib	RWE/observational	NR	94	54.3%	59.0 (31.0–84.0)
Piotrowska 2022; US [37]	1L; osimertinib	RWE/observational	November 2016–January 2022	54	29.6%	61.0
Ramalingam 2018; international [54]	1L; osimertinib	Single-arm clinical trial	4 March 2013–30 December 2022	60	25.0%	63.5 (38.0–91.0)
Bauml -a 2021; US, Canada [17]	1L+; osimertinib	RWE/observational	NR	799	32.3%	63.0 (30.0–95.0)
Le 2018; US [27,60,61]	1L+; osimertinib	RWE/observational	January 2011–February 2018	118	28.0%	63.0 (36.0–88.0)
Patil 2019; US [34]	1L+; osimertinib	RWE/observational	NR	95	33.7%	NR
Schoenfeld 2019; US [42]	1L+; osimertinib	RWE/observational	January 2016–December 2018	71	NR	NR
Ramalingam 2022; US [39]	1L or 2L; osimertinib	RWE/observational	July 2014–June 2021	2050	NR	NR
Oxnard 2018; US [33]	2L-3L; osimertinib	RWE/observational	November 2017	151	47.0%	NR
Lim 2021; US, Korea [29]	2L+; osimertinib	RWE/observational	NR	55	NR	NR
Zhao 2018; US, China [50]	2L+; osimertinib	RWE/observational	January 2017–October 2017	293	NR	NR
Guibert 2018; US [22]	Unspecified; osimertinib	RWE/observational	NR	46	NR	NR
Janne 2021; US [25,62]	Unspecified; osimertinib	RWE/observational	December 2014–November 2020	755	NR	NR
Strohbehn 2019; US [44]	Unspecified; osimertinib	RWE/observational	NR	28	NR	NR
Zhang 2018; US, China [49]	Unspecified; osimertinib	RWE/observational	NR	110	NR	NR
Osimertinib included as one treatment option among patients treated with TKIs						
Mondaca 2019; US [32]	1L; erlotinib, afatinib, gefitinib, osimertinib, rociletinib, and nazartinib	RWE/observational	January 2016–August 2017	177	35.0%	66.0 (38.0–91.0)
Robichaux 2021; US [40]	1L; EGFR TKIs (osimertinib and other TKIs)	RWE/observational	NR	16,715	NR	NR
Soria 2018; international [8,63,64]	1L; osimertinib and SoC (gefitinib and erlotinib)	Randomized clinical trial	3 December 2014–30 December 2022	556	37.1%	64.0 (2.06–93.0)
Le 2022; international [16]	1L+; tepotinib + osimertinib, and/or gefitinib	RWE/observational	NR	12	25.0%	69.5 (47.0–86.0)
Markovets 2021; international [57,65]	1L+; osimertinib + savolitinib	Non-randomized clinical trial	5 August 2014–30 December 2022	180	41.1%	61.0 (28.0–92.0)
Piotrowska 2018; US [36]	1L+; osimertinib and BLU667 + osimertinib	RWE/observational	July 2014–August 2018	41	37.0%	64.0 (40.0–87.0)
Hochmair 2018; international [24,66,67,68,69]	1L and 2L; afatinib and osimertinib	RWE/observational	28 December 2017–31 May 2018	204	46.1%	60.0 (30.0–86.0)
Rotow 2021; US [41]	2L+; osimertinib + selpercatinib	RWE/observational	NR	12	NR	NR
Goldberg 2018; US [21]	Unspecified; EGFR TKIs (afatinib, osimertinib, gefitinib, erlotinib, and rociletinib)	RWE/observational	NR	29	37.9%	60.0 (38.0–87.0)
Mack 2020; US [30]	Unspecified; EGFR TKIs (erlotinib, afatinib, gefitinib, osimertinib, rociletinib, and others)	RWE/observational	June 2014–October 2016	8388	43.0%	NR
Yang 2021; international [46]	Unspecified; afatinib post-osimertinib	RWE/observational	NR	1023	NR	NR
Yao 2019; US [47]	Unspecified; EGFR TKIs (gefitinib, osimertinib, Lenvatinib, or other EGFR TKIs)	RWE/observational	2016–2018	3600	NR	NR
Yu 2022; US [48]	Unspecified; gefitinib, afatinib, erlotinib, or osimertinib	RWE/observational	1 August 2005–1 August 2020	9	44.4%	60.0
Osimertinib included as one treatment option in patients treated with TKIs and/or non-TKIs						
Mambetsariev 2022; US [31]	1L+; erlotinib, osimertinib, afatinib, carboplatin/pemetrexed, and carboplatin/pemetrexed/pembrolizumab	RWE/observational	2014–2021	9	44.4%	60.0 (35.0–72.0)
Roper 2020; US [58,70,71,72]	1L or 2L; osimertinib and local ablative therapy (LAT)	Non-randomized clinical trial	13 April 2016–20 September 2022	34	NR	NR
Papadimitrakopoulou 2018; international [59,73]	2L; osimertinib and platinum-based doublet chemotherapy	Randomized clinical trial	4 August 2014–15 April 2016	419	35.8%	Mean (SD): 61.7 (11.7)
Patil 2020; US [35]	Unspecified; EGFR TKIs: erlotinib, gefitinib, afatinib, dacomitinib, and osimertinib Immune checkpoint inhibitors: pembrolizumab, nivolumab, or atezolizumab as single agents or in combination with chemotherapy	RWE/observational	June 2009–March 2019	570	NR	NR
Schrock 2018; US [43]	Unspecified; with or without EGFR TKIs (erlotinib, ASP8273 or afatinib + cetuximab or afatinib or erlotinib, and osimertinib)	RWE/observational	June 2012–October 2017	31	38.7%	64.0 (46.0–77.0)
Other TKIs						
Elamin 2022; US [51,74]	1L+; poziotinib	Single-arm clinical trial	17 March 2017–1 March 2023	50	40.0%	62.0 (29.0–77.0)
Helman 2018; international [23]	1L+; rociletinib	RWE/observational	Enrolled as of 1 July 2015	77	28.6%	61.0 (37.0–82.0)
Lu 2021; US [52]	1L+; aumolertinib	Single-arm clinical trial	8 May 2017–November 2022	244	NR	NR
McCoach 2021; US [53]	Unspecified; capmatinib + erlotinib	Single-arm clinical trial	2013 to 2020	35	40.0%	65.0 (39.0–89.0)
Other TKIs with non-TKIs						
Bauml-b 2021; international [55,75]	2L and 3L; amivantamab + lazertinib	Non-randomized clinical trial	24 May 2016–26 January 2024	161	NR	NR
Non-TKIs						
Janne 2022; international [56]	1L+; patritumab deruxtecan (HER3-DXd)	Non-randomized clinical trial	30 October 2017–31 December 2023	81	35.8%	64 (40–80)
Treatment/therapy name unspecified						
Gaut 2018; US [20]	1L or 2L; TKIs and chemotherapy	RWE/observational	April 2012–October 2016	97	28.9%	Mean: 66.7
Chiang 2020; US [19]	1L or 2L+; 1st- and 2nd-generation EGFR TKIs	RWE/observational	1 November 2015-30 September 2017	782	36.4%	69.0
Jin 2019; international [26,76]	Unspecified; EGFR TKIs	RWE/observational	NR	64	NR	NR
Li 2019; US [28]	Unspecified; NR	RWE/observational	January 2015–December 2015	136	85.5%	68.0 (23.0–85.0)
Raez 2022; US [38,77]	Unspecified; EGFR TKIs	RWE/observational	NR	3223	NR	NR
Suero-Abreu 2018; US [45]	Unspecified; NR	RWE/observational	September 2015–January2018	115	43.0%	68.0

Abbreviations: 1L: first-line therapy; 1L+: first-line therapy and beyond; 2L: second-line therapy; 2L+: second-line therapy and beyond; 3L: third-line therapy; EGFR: epidermal growth factor receptor; LoT: line of therapy; NR: not reported; RWE: real-world evidence; SoC: standard of care; TKI: tyrosine kinase inhibitor; US: United States.

## Data Availability

The original contributions presented in this study are included in the article/Supplementary Material. Further inquiries can be directed to the corresponding author.

## Supplementary Materials

The following supporting information can be downloaded at: https://www.mdpi.com/article/10.3390/curroncol32040191/s1, Figure S1: Summary of the Cochrane Risk of Bias Assessment for Randomized Trials (RoB2). Table S1: Search strategy for OVID-based searches. Table S2: Newcastle-Ottawa scale assessment for non-randomized studies. Table S3: Study and patient characteristics (continuation). Table S4: Resistance mutation profile for patients who received first-line osimertinib. Table S5: Resistance mutation profile for patients who received second-line osimertinib. Table S6: Resistance mutation profile for patients who received first-line osimertinib and beyond. Table S7: Resistance mutation profile for patients who received second-line osimertinib and beyond. Table S8: Resistance mutation profile for patients who received TKIs or non-TKIs, where osimertinib was one of the treatment options. Table S9: Resistance mutation profile for patients who received other TKIs or non-TKIs. Table S10: Resistance mutation profile for patients who received unspecified treatment. Table S11: Studies that reported the impact of acquired resistance on clinical outcomes. References [8,16,17,18,19,20,21,22,23,24,25,26,27,28,29,30,31,32,33,34,35,36,37,38,39,40,41,42,43,44,45,46,47,48,49,50,51,52,53,54,55,56,57,58,59] are cited in the supplementary materials.

## References

[1] Ganti A.K., Klein A.B., Cotarla I., Seal B., Chou E. (2021). Update of incidence, prevalence, survival, and initial treatment in patients with non–small cell lung cancer in the US. JAMA Oncol..

[2] Duma N., Santana-Davila R., Molina J.R. (2019). Non–small cell lung cancer: Epidemiology, screening, diagnosis, and treatment. Mayo Clin. Proc..

[3] Society A.C. Can Lung Cancer Be Found Early?. https://www.cancer.org/cancer/lung-cancer/detection-diagnosis-staging/detection.html.

[4] Jordan E.J., Kim H.R., Arcila M.E., Barron D., Chakravarty D., Gao J., Chang M.T., Ni A., Kundra R., Jonsson P. (2017). Prospective comprehensive molecular characterization of lung adenocarcinomas for efficient patient matching to approved and emerging therapies. Cancer Discov..

[5] Lindeman N.I., Cagle P.T., Aisner D.L., Arcila M.E., Beasley M.B., Bernicker E.H., Colasacco C., Dacic S., Hirsch F.R., Kerr K. (2018). Updated molecular testing guideline for the selection of lung cancer patients for treatment with targeted tyrosine kinase inhibitors: Guideline from the College of American Pathologists, the International Association for the Study of Lung Cancer, and the Association for Molecular Pathology. Arch. Pathol. Lab. Med..

[6] Tulpule A., Bivona T.G. (2020). Acquired resistance in lung cancer. Annu. Rev. Cancer Biol..

[7] Park K., Tan E.-H., O’Byrne K., Zhang L., Boyer M., Mok T., Hirsh V., Yang J.C.-H., Lee K.H., Lu S. (2016). Afatinib versus gefitinib as first-line treatment of patients with EGFR mutation-positive non-small-cell lung cancer (LUX-Lung 7): A phase 2B, open-label, randomised controlled trial. Lancet Oncol..

[8] Soria J.-C., Ohe Y., Vansteenkiste J., Reungwetwattana T., Chewaskulyong B., Lee K.H., Dechaphunkul A., Imamura F., Nogami N., Kurata T. (2018). Osimertinib in untreated EGFR-mutated advanced non–small-cell lung cancer. N. Engl. J. Med..

[9] Wu Y.-L., Cheng Y., Zhou X., Lee K.H., Nakagawa K., Niho S., Tsuji F., Linke R., Rosell R., Corral J. (2017). Dacomitinib versus gefitinib as first-line treatment for patients with EGFR-mutation-positive non-small-cell lung cancer (ARCHER 1050): A randomised, open-label, phase 3 trial. Lancet Oncol..

[10] Ramalingam S.S., Vansteenkiste J., Planchard D., Cho B.C., Gray J.E., Ohe Y., Zhou C., Reungwetwattana T., Cheng Y., Chewaskulyong B. (2020). Overall survival with osimertinib in untreated, EGFR-mutated advanced NSCLC. N. Engl. J. Med..

[11] Koulouris A., Tsagkaris C., Corriero A.C., Metro G., Mountzios G. (2022). Resistance to TKIs in EGFR-mutated non-small cell lung cancer: From mechanisms to new therapeutic strategies. Cancers.

[12] Higgins J.P.T., Thomas J., Chandler J., Cumpston M., Li T., Page M.J., Welch V.A. (2022). Cochrane Handbook for Systematic Reviews of Interventions, Version 6.3. www.training.cochrane.org/handbook.

[13] Moher D., Liberati A., Tetzlaff J., Altman D.G., PRISMA Group (2009). Preferred reporting items for systematic reviews and meta-analyses: The PRISMA statement. Ann. Intern. Med..

[14] Wells G.A., Shea B., O’Connell D., Peterson J., Welch V., Losos M., Tugwell P. (2000). The Newcastle-Ottawa Scale (NOS) for Assessing the Quality of Nonrandomised Studies in Meta-Analyses.

[15] Sterne J.A., Savović J., Page M.J., Elbers R.G., Blencowe N.S., Boutron I., Cates C.J., Cheng H.-Y., Corbett M.S., Eldridge S.M. (2019). RoB 2: A revised tool for assessing risk of bias in randomised trials. BMJ.

[16] Ahmad A., Tho L., Chik Y., Lee W., Yang T., Le X., Eisert A., Himpe U., De Bondt C., Mazieres J. (2022). 364P Tepotinib with an EGFR-tyrosine kinase inhibitor (TKI) in patients with EGFR-mutant MET-amplified NSCLC: A case series. Ann. Oncol..

[17] Bauml J., Mick R., Mccoach C., Weiss J., Marrone K., Nieva J., Villaruz L., Levy B., Moreno R., Murkherji R. (2021). FP14. 06 multicenter analysis of mechanisms of resistance to osimertinib (O) in EGFR mutated NSCLC: An ATOMIC registry study. J. Thorac. Oncol..

[18] Cardona A.F., Ruiz-Patiño A., Recondo G., Martín C., Raez L., Samtani S., Minata J.N., Blaquier J.B., Enrico D., Burotto M. (2022). Mechanisms of resistance to first-line osimertinib in Hispanic patients with EGFR mutant non-small cell lung cancer (FRESTON-CLICaP). Clin. Lung Cancer.

[19] Chiang A.C., Fernandes A.W., Pavilack M., Wu J.W., Laliberté F., Duh M.S., Chehab N., Subramanian J. (2020). EGFR mutation testing and treatment decisions in patients progressing on first-or second-generation epidermal growth factor receptor tyrosine kinase inhibitors. BMC Cancer.

[20] Gaut D., Sim M.S., Yue Y., Wolf B.R., Abarca P.A., Carroll J.M., Goldman J.W., Garon E.B. (2018). Clinical implications of the T790M mutation in disease characteristics and treatment response in patients with epidermal growth factor receptor (EGFR)-mutated non–small-cell lung cancer (NSCLC). Clin. Lung Cancer.

[21] Goldberg M.E., Montesion M., Young L., Suh J., Greenbowe J., Kennedy M., Giaccone G., Akerley W.L., Dowlati A., Creelan B.C. (2018). Multiple configurations of EGFR exon 20 resistance mutations after first-and third-generation EGFR TKI treatment affect treatment options in NSCLC. PLoS ONE.

[22] Guibert N., Hu Y., Feeney N., Kuang Y., Plagnol V., Jones G., Howarth K., Beeler J., Paweletz C., Oxnard G. (2018). Amplicon-based next-generation sequencing of plasma cell-free DNA for detection of driver and resistance mutations in advanced non-small cell lung cancer. Ann. Oncol..

[23] Helman E., Nguyen M., Karlovich C.A., Despain D., Choquette A.K., Spira A.I., Helena A.Y., Camidge D.R., Harding T.C., Lanman R.B. (2018). Cell-free DNA next-generation sequencing prediction of response and resistance to third-generation EGFR inhibitor. Clin. Lung Cancer.

[24] Hochmair M.J., Morabito A., Hao D., Yang C.-T., Soo R.A., Yang J.C., Gucalp R., Halmos B., Wang L., Golembesky A. (2018). Sequential treatment with afatinib and osimertinib in patients with EGFR mutation-positive non-small-cell lung cancer: An observational study. Future Oncol..

[25] Janne P.A., Lee J.K., Madison R., Venstrom J.M., Schrock A.B., Oxnard G.R. (2021). Incidence and heterogeneity of C797S and other EGFR resistance mutations on routine comprehensive genomic profiling (CGP). J. Clin. Oncol..

[26] Jin Y., Bao H., Le X., Fan X., Tang M., Fan Y., Zhang Y., Shi X., Zhao J., Lou G. (2019). P1.14-17 Genomic Evolution During TKI Treatment in Non-Small Cell Lung Cancer Patients With or Without Acquired T790M Mutation. J. Thorac. Oncol..

[27] Le X., Puri S., Negrao M.V., Nilsson M.B., Robichaux J., Boyle T., Hicks J.K., Lovinger K.L., Roarty E., Rinsurongkawong W. (2018). Landscape of EGFR-dependent and-independent resistance mechanisms to osimertinib and continuation therapy beyond progression in EGFR-mutant NSCLC. Clin. Cancer Res..

[28] Li B., Janku F., Jung B., Hou C., Madwani K., Alden R., Razavi P., Reis-Filho J., Shen R., Isbell J. (2019). Ultra-deep next-generation sequencing of plasma cell-free DNA in patients with advanced lung cancers: Results from the Actionable Genome Consortium. Ann. Oncol..

[29] Lim S.M., Yang S., Lim S., Heo S.G., Daniel S., Markovets A., Rafati M., Park C., Yun J., Pyo K. (2021). P76.18 Tissue-and Plasma-Based Landscape of Resistance to Osimertinib. J. Thorac. Oncol..

[30] Mack P.C., Banks K.C., Espenschied C.R., Burich R.A., Zill O.A., Lee C.E., Riess J.W., Mortimer S.A., Talasaz A., Lanman R.B. (2020). Spectrum of driver mutations and clinical impact of circulating tumor DNA analysis in non–small cell lung cancer: Analysis of over 8000 cases. Cancer.

[31] Mambetsariev I., Arvanitis L., Fricke J., Pharaon R., Baroz A.R., Afkhami M., Koczywas M., Massarelli E., Salgia R. (2022). Small cell lung cancer transformation following treatment in EGFR-mutated non-small cell lung cancer. J. Clin. Med..

[32] Mondaca S., Offin M., Borsu L., Myers M., Josyula S., Makhnin A., Shen R., Riely G.J., Rudin C.M., Ladanyi M. (2019). Lessons learned from routine, targeted assessment of liquid biopsies for EGFR T790M resistance mutation in patients with EGFR mutant lung cancers. Acta Oncol..

[33] Oxnard G.R., Hu Y., Mileham K.F., Husain H., Costa D.B., Tracy P., Feeney N., Sholl L.M., Dahlberg S.E., Redig A.J. (2018). Assessment of resistance mechanisms and clinical implications in patients with EGFR T790M–positive lung cancer and acquired resistance to osimertinib. Jama Oncol..

[34] Patil T., Dimou A., Pacheco J., Smith D., Aisner D., Merrick D., Rusthoven C., Kavanaugh B., Miller R., Schenk E. (2019). P1. 01-87 Osimertinib Acquired Resistance Mechanisms and Post-Progression Outcomes in Stage IV EGFR Positive Non-Small Lung Cancer. J. Thorac. Oncol..

[35] Patil T., Mushtaq R., Marsh S., Azelby C., Pujara M., Davies K.D., Aisner D.L., Purcell W.T., Schenk E.L., Pacheco J.M. (2020). Clinicopathologic Characteristics, Treatment Outcomes, and Acquired Resistance Patterns of Atypical EGFR Mutations and HER2 Alterations in Stage IV Non–Small-Cell Lung Cancer. Clin. Lung Cancer.

[36] Piotrowska Z., Isozaki H., Lennerz J.K., Gainor J.F., Lennes I.T., Zhu V.W., Marcoux N., Banwait M.K., Digumarthy S.R., Su W. (2018). Landscape of acquired resistance to osimertinib in EGFR-mutant NSCLC and clinical validation of combined EGFR and RET inhibition with osimertinib and BLU-667 for acquired RET fusion. Cancer Discov..

[37] Piotrowska Z., Piper-Vallillo A., Banwait M., Hung Y.P., Rao R., Muzikansky A., Meador C.B., Hata A.N., Sequist L.V. (2022). Complete evaluation of resistance mechanisms to first-line osimertinib requires tissue biopsy. J. Clin. Oncol..

[38] Raez L.E., Baca Y., Nieva J.J., Mamdani H., Lopes G., Borghaei H., Socinski M.A., Nabhan C., Wozniak A.J., Vanderwalde A.M. (2022). Acquired EGFR-resistant mutations in non–small cell lung cancer (NSCLC). J. Clin. Oncol..

[39] Ramalingam S., Zhang N., Yu J., Espenschied C., Green T., Infantine J., Mar B. (2022). MA07. 03 Real-world Landscape of EGFR C797X Mutation as a Resistance Mechanism to Osimertinib in Non-small Cell Lung Cancer. J. Thorac. Oncol..

[40] Robichaux J., Le X., Vijayan R., Hicks K., Elamin Y., Tran H., Varghese S., He J., Zhang F., Hu L. (2021). MA13. 07 Structural Classification of Atypical EGFR Mutations Identifies 4 Major Subgroups With Distinct Patterns of Drug Sensitivity. J. Thorac. Oncol..

[41] Rotow J., Patel J., Hanley M., Yu H., Goldman J., Nechustan H., Scheffler M., Awad M., Clifford S., Santucci A. (2021). FP14. 07 combination osimertinib plus selpercatinib for EGFR-mutant non-small cell lung cancer (NSCLC) with acquired RET fusions. J. Thorac. Oncol..

[42] Schoenfeld A.J., Chan J.M., Rizvi H., Rekhtman N., Daneshbod Y., Kubota D., Chang J.C., Arcila M.E., Ladanyi M., Somwar R. (2019). Tissue-based molecular and histological landscape of acquired resistance to osimertinib given initially or at relapse in patients with EGFR-mutant lung cancers. J. Clin. Oncol..

[43] Schrock A.B., Zhu V.W., Hsieh W.-S., Madison R., Creelan B., Silberberg J., Costin D., Bharne A., Bonta I., Bosemani T. (2018). Receptor tyrosine kinase fusions and BRAF kinase fusions are rare but actionable resistance mechanisms to EGFR tyrosine kinase inhibitors. J. Thorac. Oncol..

[44] Strohbehn G., Szeto L., Beach B., Edgington K., Lugtu K., Segal J., Ritterhouse L., Bestvina C., Vokes E., Patel J. (2019). P2. 14-12 Tyrosine Kinase Inhibitor Resistance Mechanisms in EGFR T790M-Positive Lung Cancer: The University of Chicago Experience. J. Thorac. Oncol..

[45] Suero-Abreu G.A., Gonzalez Velez M., Proverbs-Singh T.A., Gutierrez M. (2019). Circulating tumor DNA (ctDNA) for genomic profiling of non-small cell lung cancer (NSCLC): Experience in a large community-based cancer center. J. Clin. Oncol..

[46] Yang J.-H., Schuler M., Popat S., Miura S., Park K., Passaro A., De Marinis F., Solca F., Märten A., Kim E. (2021). 1212P Afatinib for the treatment of NSCLC with uncommon EGFR mutations: An updated database of 1023 cases. Ann. Oncol..

[47] Yao Y., Zhang M., Liu X., Zhao J., Cheng X., Zeng A., Kong J., Zhang H., Chen R., Xia X. (2019). RET fusion in first/third-generation EGFR-TKIs resistance in advanced non-small cell lung cancer. J. Clin. Oncol..

[48] Yu L., Bazhenova L., Gold K., Tran L., Hilburn V., Vu P., Patel S.P. (2022). Clinicopathologic and molecular characteristics of EGFR-mutant lung adenocarcinomas that transform to small cell lung cancer after TKI therapy. Transl. Lung Cancer Res..

[49] Zhang Y., Zhao J., Guo R., Lin G., Liu L., Zhu C., Liang N., Yang H., Wang W.X., Dai P. (2018). Landscape of osimertinib resistant mutations between the two common subtypes of EGFR 19del or L858R in NSCLC. J. Clin. Oncol..

[50] Zhao J., Chen R., Lin G., Ai X., Sheng W., Ji Y., Fan Z., Miao L., Zhu L., Zhao Q. (2018). Next generation sequencing (NGS) based mutation profiling and heterogeneity of resistance mechanisms to AZD9291. J. Clin. Oncol..

[51] Elamin Y.Y., Robichaux J.P., Carter B.W., Altan M., Tran H., Gibbons D.L., Heeke S., Fossella F.V., Lam V.K., Le X. (2022). Poziotinib for EGFR exon 20-mutant NSCLC: Clinical efficacy, resistance mechanisms, and impact of insertion location on drug sensitivity. Cancer Cell.

[52] Lu S., Wang Q., Zhang G., Dong X., Yang C., Song Y., Chang G., Lu Y., Pan H., Chiu C. (2021). 1208P Final results of APOLLO study: Overall survival (OS) of aumolertinib in patients with pretreated EGFR T790M-positive locally advanced or metastatic non-small cell lung cancer (NSCLC). Ann. Oncol..

[53] McCoach C.E., Yu A., Gandara D.R., Riess J.W., Vang D.P., Li T., Lara P.N., Gubens M., Lara F., Mack P.C. (2021). Phase I/II study of capmatinib plus erlotinib in patients with MET-positive non–small-cell lung cancer. Jco Precis. Oncol..

[54] Ramalingam S.S., Yang J., Lee C.K., Kurata T., Kim D.-W., John T., Nogami N., Ohe Y., Mann H., Rukazenkov Y. (2018). Osimertinib as first-line treatment of EGFR mutation-positive advanced non-small-cell lung cancer. J. Clin. Oncol..

[55] Bauml J., Cho B.C., Park K., Lee K.H., Cho E.K., Kim D.-W., Kim S.-W., Haura E.B., Sabari J.K., Sanborn R.E. (2021). Amivantamab in combination with lazertinib for the treatment of osimertinib-relapsed, chemotherapy-naïve EGFR mutant (EGFRm) non-small cell lung cancer (NSCLC) and potential biomarkers for response. J. Clin. Oncol..

[56] Jänne P.A., Baik C., Su W.-C., Johnson M.L., Hayashi H., Nishio M., Kim D.-W., Koczywas M., Gold K.A., Steuer C.E. (2022). Efficacy and safety of patritumab deruxtecan (HER3-DXd) in EGFR inhibitor–resistant, EGFR-mutated non–small cell lung cancer. Cancer Discov..

[57] Markovets A., Han J.-Y., Cho B.C., Cantarini M., Janne P.A., Hartmaier R. (2021). Acquired resistance in patients with EGFRm NSCLC following treatment with osimertinib plus savolitinib in the Ph1b TATTON study Parts B and D. Cancer Res..

[58] Roper N., Brown A.-L., Wei J.S., Pack S., Trindade C., Kim C., Restifo O., Gao S., Sindiri S., Mehrabadi F. (2020). Clonal evolution and heterogeneity of osimertinib acquired resistance mechanisms in EGFR mutant lung cancer. Cell Rep. Med..

[59] Papadimitrakopoulou V., Wu Y.-L., Han J.-Y., Ahn M.-J., Ramalingam S., John T., Okamoto I., Yang J.-H., Bulusu K., Laus G. (2018). Analysis of resistance mechanisms to osimertinib in patients with EGFR T790M advanced NSCLC from the AURA3 study. Ann. Oncol..

[60] Le X., Puri S., Negrao M.V., Nilsson M.B., Robichaux J.P., Boyle T.A., Hicks J.K., Roarty E., Rinsurongkawong W., Glisson B.S. (2018). Landscape of EGFR-dependent and independent mechanisms of osimertinib resistance in EGFR-mutant NSCLC patients. J. Clin. Oncol..

[61] Le X., Negrao M.V., Nilsson M., Robichaux J., Roarty E., Rinsurongkawong W., Glisson B., Zhang J., Heymach J.V. (2018). Mechanisms of resistance for osimertinib for patients with EGFR-mutant lung cancer: MD Anderson Cancer Center single institution experience with osimertinib resistance. Cancer Res..

[62] Yu H.A., Baik C.S., Gold K., Hayashi H., Johnson M., Koczywas M., Murakami H., Nishio M., Steuer C., Su W.C. (2020). Efficacy and safety of patritumab deruxtecan (U3-1402), a novel HER3 directed antibody drug conjugate, in patients (pts) with EGFR-mutated (EGFRm) NSCLC. Ann. Oncol..

[63] Cho B.C., Cheng Y., Zhou C., Ohe Y., Imamura F., Lin M.C., Majem M., Shah R., Rukazenkov Y., Todd A. (2018). LBA8—Mechanisms of acquired resistance to first-line osimertinib: Preliminary data from the phase III FLAURA study. Ann. Oncol..

[64] Ramalingam S.S., Cheng Y., Zhou C., Ohe Y., Imamura F., Cho B.C., Lin M.C., Majem M., Shah R., Rukazenkov Y. (2018). LBA50—Mechanisms of acquired resistance to first-line osimertinib: Preliminary data from the phase III FLAURA study. Ann. Oncol..

[65] Sequist L.V., Han J.-Y., Ahn M.-J., Cho B.C., Yu H., Kim S.-W., Yang J.C.-H., Lee J.S., Su W.-C., Kowalski D. (2020). Osimertinib plus savolitinib in patients with EGFR mutation-positive, MET-amplified, non-small-cell lung cancer after progression on EGFR tyrosine kinase inhibitors: Interim results from a multicentre, open-label, phase 1b study. Lancet. Oncol..

[66] Halmos B., Hochmair M.J., Morabito A., Hao D., Yang C.T., Soo R.A., Yang J.C.H., Gucalp R., Wang L., Marten A. (2019). Afatinib followed by osimertinib in EGFR mutation-positive (EGFRM+) advanced NSCLC: Subgroup analyses of the giotag study by ECOG PS, age, and ethnicity. Jnccn J. Natl. Compr. Cancer Netw..

[67] Hochmair M.J., Morabito A., Hao D., Yang C.T., Soo R., Yang J.C.H., Gucalp R., Halmos B., Wang L., Golembesky A. (2019). Afatinib followed by osimertinib in patients with EGFR mutation-positive (EGFRm+) advanced NSCLC: Updated data from the GioTag real-world study. Ann. Oncol..

[68] Hochmair M.J., Morabito A., Hao D., Yang C.T., Soo R.A., Yang J.C.H., Gucalp R., Halmos B., Wang L., Golembesky A. (2018). Afatinib followed by osimertinib in patients with EGFR mutation-positive advanced NSCLC: A real-world study (GioTag). Ann. Oncol..

[69] Hochmair M.J., Morabito A., Hao D., Yang C., Soo R., Yang J.C., Gucalp R., Halmos B., Golembesky A., Marten A. (2018). Afatinib Followed by Osimertinib in Real-World Patients with EGFR Mutation-Positive Advanced NSCLC: The Giotag Study. J. Thorac. Oncol..

[70] Kim C., Xi L., Cultraro C.M., Pham T.H.T., Shafei A., Roper N., Bagheri M., Beeler J., Jones G., Raffeld M. (2018). Circulating Tumor DNA Analysis for Predicting Response to Osimertinib and Disease Progression in EGFR-Mutant Non-Small-Cell Lung Cancer. J. Thorac. Oncol..

[71] Kim C., Roper N., Hoang C., Wisch L., Connolly M., Chou H.C., Wei J., Tyagi M., Cultraro C.M., Xi L. (2018). Local ablative therapy for oligoprogressive, EGFR-mutant, non-small cell lung cancer (NSCLC) after treatment with osimertinib. Cancer Res..

[72] Kim C., Xi L., Cultraro C., Wei F., Cheng J., Shafiei A., Pham T., Roper N., Akoth E., Strom C. (2019). P1.01-27 Serial Circulating Tumor DNA (ctDNA) Analysis of Blood and Saliva Predicts Osimertinib Response and Resistance in EGFR-Mutant NSCLC. J. Thorac. Oncol..

[73] Vaclova T., Grazini U., Ward L., O’Neill D., Markovets A., Huang X., Chmielecki J., Hartmaier R., Thress K.S., Smith P.D. (2021). Clinical impact of subclonal EGFR T790M mutations in advanced-stage EGFR-mutant non-small-cell lung cancers. Nat. Commun..

[74] Elamin Y., Robichaux J., Carter B., Altan M., Gibbons D., Fossella F., Simon G., Lam V., Blumenschein G., Tsao A. (2019). MA09.03 Identification of Mechanisms of Acquired Resistance to Poziotinib in EGFR Exon 20 Mutant Non-Small Cell Lung Cancer (NSCLC). J. Thorac. Oncol..

[75] Haura E.B., Lee J.S., Han J.Y., Lee K.H., Sanborn R.E., Govindan R., Cho E.K., Kim S.W., Reckamp K.L., Sabari J.K. (2019). JNJ-61186372 (JNJ-372), an EGFR-cMet bispecific antibody, in EGFR-driven advanced non-small cell lung cancer (NSCLC). J. Clin. Oncol..

[76] Jin Y., Bao H., Le X., Fan X., Tang M., Fan Y., Zhang Y., Xu Y., Wu X., Shao Y. (2019). Distinct resistant mechanism and genomic evolution during TKI treatment in non-small cell lung cancer patients with or without acquired T790M mutation. J. Clin. Oncol..

[77] Raez L.E., Baca Y., Nagasaka M., Nieva J., Mandani H., Wanderwalde A., Borghaei H., Naban C., Langer C., Socinsky M.A. (2022). Developing of EGFR resistant mutations to Tyrosine Kinase Inhibitors (TKI) in Non-Small Cell Lung Cancer (NSCLC). J. Thorac. Oncol..

[78] Kobayashi N., Katakura S., Kamimaki C., Somekawa K., Fukuda N., Tanaka K., Watanabe K., Horita N., Hara Y., Piao H. (2021). Resistance mechanisms of epidermal growth factor receptor tyrosine kinase inhibitors in non-small cell lung cancer patients: A meta-analysis. Thorac. Cancer.

