# Supplementary material for: Resistance Mutation Profiles Associated with Current Treatments for Epidermal Growth Factor Receptor-Mutated Non-Small-Cell Lung Cancer in the United States: A Systematic Literature Review
Source: Curr Oncol. 2025 Mar 25;32(4):191. doi: 10.3390/curroncol32040191 (PMC12025648; doi:10.3390/curroncol32040191)
Supplement: Supplementary file 1 [file curroncol-32-00191-s001.zip › curroncol-3409061-supplementary.pdf]

Supplementary Figure S1. Summary of the Cochrane Risk of Bias Assessment for Randomized Trials (RoB2). [8,59].

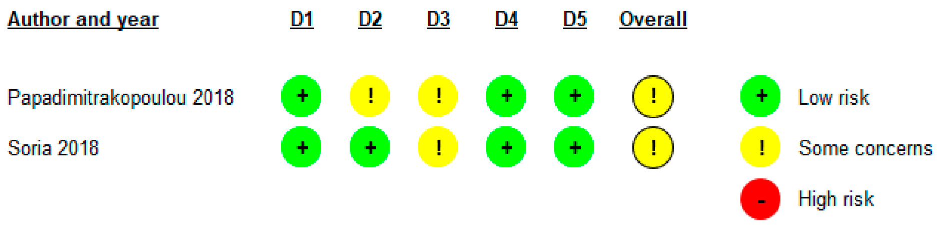

Supplementary Table S1. Search strategy for OVID-based searches.

Database(s): **Embase** 1974 to August 02, 2022, **Ovid MEDLINE(R) and Epub Ahead of Print, In-Process, In-Data-Review & Other Non-Indexed Citations and Daily** 1946 to August, 02, 2022

Searches	Results
(((nonsmall or non-small or "non small") and (lung or pulmonar*)) or NSCLC).ab,ti.	226208
(epidermal-growth-factor-receptor* or EGFR* or erbb-receptor* or erbb* or egf-receptor* or her-family).ab,ti. AND ((exp mutation/ or exp mutations/ or exp mutant protein/ or exp mutant proteins/ or (mutation or mutations or mutated or mutant or deletion or insertion).ab,ti. or (TKI or "tyrosine kinase inhibitor" or inhibitor* or Osimertinib).ab,ti. or ((gene* or oncogene* or molecular* or protein*) adj7 (alteration* or aberration or amplification or expression or overexpression)).ab,ti.))	158970
exp drug resistance/ or resistan*.ab,ti.	2722448
1 and 2 and 3	12760
Limit 4 to English	12352
5 not (review.ti,pt. or ("systematic review" or "systematic literature review").ab,ti,pt. or (case-report* or case-stud*).ti,pt,hw,xs,sh. or (commentary or editorial or letter).pt.)	9719
Limit 6 to yr="2018-Current"	4452
Remove duplicates from 7	3023

Supplementary Table S2. Study and patient characteristics continuation.

Author, year, study design	Study name	Publication type	Sample source	N	Population description	Treatment groups	Sex, Male %	Race/Ethnicity, %	Smoking, %	Site of metastasis, %
**Osimertinib**										
Cardona 2022 [18], Observational study	FRESTON-CLICaP	Journal article	Database at the Foundation for Clinical and Applied Cancer Research (FICMAC).	94	Hispanic patients with EGFR mutated NSCLC.	Osimertinib	54.3%	Hispanic: 100.0%	Active/former: 53.2%; Never: 46.8%	Brain: 44.7%; Lung/pleura: 76.6%; Lymph nodes: 14.9%; Liver and bone: 11.7%; Leptomeningeal: 7.4%; Adrenal glands: 4.4%
Piotrowska 2022 [37], Observational study	NA	Conference abstract	Medical charts (Massachusetts General Hospital Cancer Center).	54	Advanced EGFR+ NSCLC.	Osimertinib	29.6%	NR	NR	NR
Ramalingam 2018 [54], Single-arm clinical trial	AURA	Journal article	NA	60	Treatment naïve, EGFR-mutated advanced NSCLC.	Osimertinib	25.0%	White: 25.0%; Asian: 72.0%; Unknown: 2.0%; American Indian or Alaska native: 2.0%	NR	CNS: 25.0%
Bauml 2021-a [17], Observational study	NA	Conference abstract	Database and secure web portal by The Academic Thoracic Oncology Medical Investigator's Consortium (ATOMIC).	799	Metastatic EGFR mutated NSCLC.	Osimertinib	32.3%	White: 53.0%	NR	NR
Le 2018 [27], Observational study	NA	Journal article	University of Texas MD Anderson Lung Cancer Moon Shot GEMINI	118	Advanced EGFR-mutant NSCLC.	Osimertinib	28.0%	NR	Former: 32.0%; Never: 68.0%	CNS: 26.0%

Author, year, study design	Study name	Publication type	Sample source	N	Population description	Treatment groups	Sex, Male %	Race/Ethnicity, %	Smoking, %	Site of metastasis, %
			database, Moffitt electronic health record, Clinical Genomic Action Committee database and pyrosequencing database.							
Patil 2019 [34], Observational study	NA	Conference abstract	Medical charts of University of Colorado-Amc, Aurora.	95	Stage IV EGFR+ NSCLC.	Osimertinib	33.7%	NR	Never: 75.8%	NR
Schoenfeld 2019 [42], Observational study	NA	Conference abstract	Source unclear	71	EGFR mutant lung cancers.	Osimertinib	NR	NR	NR	NR
Ramalingam 2022 [39], Observational study	NA	Conference abstract	Guardant INFORM real-world clinical genomic database.	2,050	Metastatic NSCLC with activating EGFR mutations.	Osimertinib	NR	NR	NR	NR
Oxnard 2018 [33], Observational study	NA	Journal article	Institutional cohort: Institutional databases of 4 contributing cancer centers.<br>AURA validation cohort: Retrospective analysis of patients in the AURA trial.	151	Advanced EGFR T790M-positive NSCLC.	Osimertinib	47.0%	NR	NR	NR
Lim 2021 [29], Observational study	NA	Conference abstract	Medical charts from multiple institutes (international).	55	EGFR mutated NSCLC.	Osimertinib	NR	NR	NR	NR
Zhao 2018 [50], Observational study	NA	Conference abstract	Source unclear	293	Advanced lung adenocarcinoma patients.	Osimertinib	NR	NR	NR	NR

Author, year, study design	Study name	Publication type	Sample source	N	Population description	Treatment groups	Sex, Male %	Race/Ethnicity, %	Smoking, %	Site of metastasis, %
Guibert 2018 [22], Observational study	NA	Journal article	Source unclear	46	Stage IIIB/IV, progressive NSCLC.	Osimertinib	NR	NR	NR	NR
Janne 2021 [25], Observational study	NA	Conference abstract	Flatiron Health-Foundation Medicine clinico-genomic database (CGDB) linked to Comprehensive genomic profiling (CGP) data.	755	EGFR mutated NSCLC.	Osimertinib	NR	NR	NR	NR
Strohbehn 2019 [44], Observational study	NA	Conference abstract	University of Chicago Medicine case records.	28	Osimertinib-treated NSCLC patients who progressed through therapy.	Osimertinib	NR	NR	NR	NR
Zhang 2018 [49], Observational study	NA	Conference abstract	Source unclear	110	Advanced NSCLC patients with an EGFR activating mutation (19del or L858R) and were clinically resistant to osimertinib.	Osimertinib	NR	NR	NR	NR
**Osimertinib included as one treatment option among patients treated with TKIs**										
Mondaca 2019 [32], Observational study	NA	Journal article	Electronic medical records (Memorial Sloan Kettering Cancer Center (MSK)).	177	Metastatic EGFR-mutant NSCLC.	Erlotinib, Afatinib, Gefitinib, Osimertinib, Rociletinib, Nazartinib	35.0%	NR	Never: 53.0%	NR

Author, year, study design	Study name	Publication type	Sample source	N	Population description	Treatment groups	Sex, Male %	Race/Ethnicity, %	Smoking, %	Site of metastasis, %
Robichaux 2021 [40], Observational study	NA	Conference abstract	MD Anderson GEMINI database and Moffitt Cancer Center.	16,715	EGFR mutant NSCLC.	EGFR-TKIs (Osimertinib, Other TKIs)	NR	NR	NR	NR
Soria 2018 [8], Randomized clinical trial	FLAURA	Journal article	NA	556	Previously untreated EGFRm (tissue, ex19del/L858R) advanced NSCLC.	Osimertinib, SoC (Gefitinib, Erlotinib)	37.1%	White: 36.2%; Black: 0.7%; Asian: 62.4%; Unknown: 0.4%; American Indian or Alaska native: 0.2%	Current: 3.1%; Former: 32.7%; Never: 64.2%	Brain: 20.9%; Visceral: 35.4%
Le 2022 [16], Observational study	NA	Conference abstract	Source unclear	12	EGFR-mutant MET amplified NSCLC.	Tepotinib + Osimertinib and/or Gefitinib	25.0%	NR	NR	NR
Markovets 2021 [57], Non-randomized clinical trial	TATTON	Conference abstract	NA	180	MET-amplified EGFR mutant advanced NSCLC.	Osimertinib + Savolitinib	41.1%	White: 28.9%; Asian: 71.1%	NR	Brain: 43.3%
Piotrowska 2018 [36], Observational study	NA	Journal article	Medical charts from Massachusetts General Hospital (MGH).	41	EGFR-mutant NSCLC.	Osimertinib, BLU667 + Osimertinib	37.0%		NR	NR
Hochmair 2018 [24], Observational study	GioTag	Journal article	Sponsor or from electronic health records supplied by Cardinal Health (OH, USA).	204	TKI naive, EGFR mutation-positive advanced NSCLC.	Afatinib, Osimertinib	46.1%	White: 58.6%; Black: 8.8%; Asian: 24.5%; Hispanic: 9.3%; Unknown: 7.8%	NR	Brain: 10.3%

Author, year, study design	Study name	Publication type	Sample source	N	Population description	Treatment groups %	Sex, Male	Race/Ethnicity, % %	Smoking,	Site of metastasis, %
Rotow 2021 [41], Observational study	NA	Conference abstract	Three selpercatinib compassionate access programs: single patient protocols (SPP), named patient programs (NPPs), and an expanded access program (EAP).	12	Advanced EGFR-mutated NSCLC with a RET fusion.	Osimertinib + Selpercatinib	NR	NR	NR	NR
Goldberg 2018 [21], Observational study	NA	Journal article	Medical charts - Western Institutional Review Board (WIRB, Puyallup WA).	29	NSCLC with an EGFR mutation at codons 792, 796, or 797.	EGFR-TKIs (Afatinib, Osimertinib, Gefitinib, Erlotinib, Rociletinib)	37.9%	NR	NR	Brain: 3.4%
Mack 2020 [30], Observational study	NA	Journal article	Electronic case report forms from multiple US institutes.	8,388	Advanced (stage IIIB-IV) lung adenocarcinoma (LUAD) or NSCLC not otherwise specified (NSCLC-NOS).	EGFR-TKIs (Erlotinib, Afatinib, Gefitinib, Osimertinib, Rociletinib, Other)	43.0%	NR	NR	NR
Yang 2021 [46], Observational study	NA	Conference abstract	Prospective database developed by Boehringer Ingelheim and published cases.	1,023	NSCLC with uncommon EGFR mutations.	Afatinib post Osimertinib	NR	NR	NR	NR
Yao 2019 [47], Observational study	NA	Conference abstract	Source unclear	3,600	Advanced NSCLC.	EGFR-TKIs (Gefitinib, osimertinib, Lenvatinib or	NR	NR	NR	NR

Author, year, study design	Study name	Publication type	Sample source	N	Population description	Treatment groups	Sex, Male %	Race/Ethnicity, %	Smoking, %	Site of metastasis, %
						other EGFR TKIs)				
Yu 2022 [48], Observational study	NA	Journal article	EMR at Moores Cancer Center of the University of California.	9	EGFR-mutant lung adenocarcinoma after EGFR-TKIs treatment.	Gefitinib, Afatinib, Erlotinib or Osimertinib	44.4%	NR	Former: 55.6%; Never: 44.4%	CNS: 33.3%
**Osimertinib included as one treatment option among patients treated with TKIs and/or non-TKIs**										
Mambetsariev 2022 [31], Observational study	NA	Journal article	Electronic medical records of City of Hope.	9	EGFR-mutated lung adenocarcinoma.	Erlotinib, Osimertinib, Afatinib, Carboplatin/Pemetrexed, Carboplatin/Pemetrexed/Pembrolizumab	44.4%	White: 55.6%; Asian: 44.4%	Never: 55.6%	Liver: 33.3%
Roper 2020 [58], Non-randomized clinical trial	NA	Journal article	NA	34	Histologically confirmed EGFR mutant stage IV lung adenocarcinoma.	Osimertinib, Local ablative therapy (LAT)	NR	NR	NR	Multiple: 94.1%
Papadimitrakopoulou 2018 [59], Randomized clinical trial	AURA 3	Conference abstract	NA	419	T790M-positive advanced NSCLC, whose disease progressed on or after first-line EGFR-TKIs therapy.	Osimertinib, Platinum-based doublet chemotherapy	35.8%	White: 32.0%; Black: 1.2%; Asia: 65.4%; Unknown: 1.2%; American Indian or Alaska native: 0.2%	NR	NR
Patil 2020 [35], Observational study	NA	Journal article	Database that included all patients treated through the University of	570	Stage IV NSCLC.	EGFR-TKIs: First-generation TKIs (erlotinib,	NR	NR	NR	NR

Author, year, study design	Study name	Publication type	Sample source	N	Population description	Treatment groups	Sex, Male %	Race/Ethnicity, %	Smoking, %	Site of metastasis, %
			Colorado health system.			gefitinib), second-generation TKIs (afatinib, dacomitinib), third generation TKIs (osimertinib). Immune checkpoint inhibitors: Pembrolizumab, Nivolumab or Atezolizumab as single agents or in combination with chemotherapy (using KEYNOTE-189 regimen).				
Schrock 2018 [43], Observational study	NA	Journal article	Foundation Medicine, Inc, database.	31	NSCLC with classical activating EGFR mutations.	With or without EGFR-TKIs (Erlotinib, ASP8273 or Afatinib + Cetuximab or Afatinib or Erlotinib, Osimertinib)	38.7%	NR	NR	NR
**Other TKIs**										

Author, year, study design	Study name	Publication type	Sample source	N	Population description	Treatment groups	Sex, Male %	Race/Ethnicity, %	Smoking, %	Site of metastasis, %
Elamin 2022 [51], Single-arm clinical trial	NA	Journal article	NA	50	Histologically or cytologically confirmed, locally advanced or metastatic NSCLC (stage IIIB and IV) patients.	Poziotinib	40.0%	White: 76.0%; Black: 8.0%; Asian: 16.0%	Former: 32.0%; Never: 68.0%	Brain: 28.0%
Helman 2018 [23], Observational study	TIGER-X, TIGER-2	Journal article	Patients enrolled onto the TIGER-X (NCT01526928) or TIGER-2 (NCT02147990) studies.	77	NSCLC with or without EGFR T790M mutations.	Rociletinib	28.6%	White: 59.7%; Black: 6.5%; Asian: 20.8%; Unknown: 13.0%	NR	Brain: 40.3%
Lu 2021 [52], Single-arm clinical trial	APOLLO	Conference abstract	NA	244	Pretreated EGFR T790M-positive locally advanced or metastatic NSCLC.	Aumolertinib	NR	NR	NR	NR
McCoach 2021 [53], Single-arm clinical trial	NA	Journal article	NA	35	Advanced /metastatic NSCLC patients.	Capmatinib + Erlotinib	40.0%	White: 80.0% Asian: 17.1% Hispanic: 8.6% Unknown: 2.9%	NR	NR
**Other TKIs with non-TKIs**										
Bauml 2021-b [55], Non-randomized clinical trial	CHRYSALIS	Conference abstract	NA	161	EGFR exon 19deletion or L858R mutation NSCLC who had relapsed on osimertinib	Amivantamab + Lazertinib	NR	Asian: 77.0%	NR	NR

Author, year, study design	Study name	Publication type	Sample source	N	Population description	Treatment groups	Sex, Male %	Race/Ethnicity, %	Smoking, %	Site of metastasis, %
					or 3rd generation TKI-relapsed EGFR mutant NSCLC and EGFR Exon20ins disease.					
**Non-TKIs**										
Janne 2022 [56], Non-randomized clinical trial	U31402-A-U102	Journal article	NA	81	Locally advanced or metastatic EGFR-mutated NSCLC and prior EGFR-TKI therapy.	Patritumab deruxtecan (HER3-DXd)	35.8%	NR	NR	Brain: 21.0%
**Treatment/therapy name unspecified**										
Gaut 2018 [20], Observational study	NA	Journal article	Medical records acquired from enrolled and screen-failed patients for the clinical trials NCT02147990 (TIGER-2) and NCT01526928 (TIGER-X) involving the third generation TKI rociletinib (CO-1686).	97	Confirmed metastatic or unresectable locally advanced EGFR-mutant NSCLC.	TKIs, Chemotherapy	28.9%	White: 24.7%; Black: 1.0%; Asian: 26.8%; Hispanic: 7.2%; Unknown: 40.2%	Current or former Smoker: 22.7%; Never: 77.3%	NR
Chiang 2020 [19], Observational study	NA	Journal article	Flatiron Health electronic health records-derived database.	782	Metastatic NSCLC upon progression on first-/second-generation EGFR-TKIs.	1st and 2nd generations EGFR-TKIs	36.4%	White: 56.3%; Black: 9.3%; Asian: 8.7%; Unknown: 25.7%	Never: 36.7%; Unknown: 0.3%	NR

Author, year, study design	Study name	Publication type	Sample source	N	Population description	Treatment groups	Sex, Male %	Race/Ethnicity, %	Smoking, %	Site of metastasis, %
Jin 2019 [26], Observational study	NA	Conference abstract	Source unclear.	64	EGFR mutant NSCLC with or without acquired T790M mutation.	EGFR-TKIs	NR	NR	NR	NR
Li 2019 [28], Observational study	NA	Journal article	Medical charts (MSK Cancer Center, MD Anderson Cancer Center, and Dana- Farber Cancer Center).	136	Newly diagnosed, progressing stage IV, or recurrent metastatic NSCLC.	NR	85.5%	NR	NR	NR
Raez 2022 [38], Observational study	NA	Conference abstract	Treatment from insurance claims data, sequencing data from Caris Life Sciences.	3,223	EGFR mutant NSCLC.	EGFR-TKIs	NR	NR	NR	NR
Suero-Abreu 2018 [45], Observational study	NA	Conference abstract	Medical charts of community-based cancer center in New Jersey.	115	Patients with NSCLC.	NR	43.0%	White: 65.0%; Black: 14.0%; Hispanic: 16.0%; Unknown: 5.0%	Current: 11.0%; Former: 56.0%; Never: 33.0%	NR

Supplementary Table S3. Newcastle-Ottawa Scale Assessment for Non-randomized Studies

Studies reporting resistance mutation profile and clinical outcomes (max score = 6)[a]

Author and year	Selection			Outcomes			NOS Score
	Representativeness of the exposed cohort	Ascertainment of exposure	Demonstration that outcome of interest was not present at start of study	Assessment of outcome	Was follow-up long enough for outcomes to occur	Adequacy of follow up of cohorts	Total stars (max star = 6)
Bauml 2021-b [55]		*		*	*	*	4
Cardona 2022 [18]	*	*		*			3
Gaut 2018 [20]		*		*		*	3
Helman 2018 [23]			*	*		*	3
Janne 2022 [56]	*	*		*	*	*	5
Jin 2019 [26]			*	*			2
Le 2022 [16]				*		*	2
McCoach 2021 [53]	*			*			2
Mondaca 2019 [32]	*			*		*	3
Oxnard 2018 [33]				*		*	2
Robichaux 2021 [40]	*						1
Schrock 2018 [43]	*		*	*			3
Yang 2021 [46]	*						1
Yao 2019 [47]	*			*			2

[a] The following domains for NOS were considered not applicable in this case, and therefore not evaluated or presented in the table: selection of the non-exposed cohort (1 star), comparability of cohorts on the basis of the design or analysis (2 stars).

Studies reporting only resistance mutation profile (max score = 4) [b]

Author and year	Selection			Outcomes	NOS Score
	Representativeness of the exposed cohort	Ascertainment of exposure	Demonstration that outcome of interest was not present at start of study	Assessment of outcome	Total stars (max star = 4)
Bauml 2021-a [17]	*		*	*	3
Chiang 2020 [19]	*	*	*	*	4
Elamin 2022 [51]		*	*	*	3
Goldberg 2018 [21]				*	1
Guibert 2018 [22]	*		*	*	3
Hochmair 2018 [24]				*	1
Janne 2021 [25]	*		*	*	3
Le 2018 [27]				*	1

Author and year	Selection			Outcomes	NOS Score
	Representativeness of the exposed cohort	Ascertainment of exposure	Demonstration that outcome of interest was not present at start of study	Assessment of outcome	Total stars (max star = 4)
Li 2019 [28]	*			*	2
Lim 2021 [29]	*		*	*	3
Lu 2021 [52]		*		*	2
Mack 2020 [30]	*	*		*	3
Mambetsariev 2022 [31]	*	*		*	3
Markovets 2021 [57]		*	*	*	3
Patil 2019 [34]	*		*	*	3
Patil 2020 [35]	*	*		*	3
Piotrowska 2022 [37]	*			*	2
Piotrowska 2018 [36]	*	*		*	3
Raez 2022 [38]	*			*	2
Ramalingam 2022 [39]	*			*	2
Ramalingam 2018 [54]	*	*	*	*	4
Roper 2020 [58]	*	*	*	*	4
Rotow 2021 [41]				*	1
Schoenfeld 2019 [42]	*		*	*	3
Strohbehn 2019 [44]	*			*	2
Suero-Abreu 2018 [45]	*			*	2
Yu 2022 [48]	*		*	*	3
Zhang 2018 [49]				*	1
Zhao 2018 [50]				*	1

[b] The following items for NOS were considered not applicable in this case, and therefore not evaluated or presented in the table: selection of the non-exposed cohort (1 star), comparability of cohorts on the basis of the design or analysis (2 stars), was follow-up long enough for outcomes to occur (1 star), adequacy of follow up of cohorts (1 star).

Supplementary Table S4. Resistance mutation profile for patients who received first-line osimertinib

Gene name	Resistance mechanism	Author, year	Sample size	N (%) with acquired resistance
**EGFR-dependent mechanisms**				
EGFR	Acquired mutations	Cardona 2022 [18]	147	17 (11.6)
	Amplification	Cardona 2022 [18]	147	12 (8.4)
		Piotrowska 2022 [37]	35[a]	1 (2.9) - tissue biopsy
		Piotrowska 2022 [37]	32[a]	2 (6.0) - liquid biopsy

Gene name	Resistance mechanism	Author, year	Sample size	N (%) with acquired resistance
	C797S mutation	Piotrowska 2022 [37]	35[a]	1 (2.9) - tissue biopsy
		Piotrowska 2022 [37]	32[a]	3 (9.4) - liquid biopsy
		Ramalingam 2018 [54]	19	2 (10.5)
		Soria 2018 [8]	91	6 (7.0)
	C797S, G724S and L718V mutation	Piotrowska 2022 [37]	32	1 (3.1) - liquid biopsy
	C797X mutation	Ramalingam 2022 [39]	1,337	107 (8.0)- 5 years after osimertinib initiation
			1,337	87 (6.5) - at median follow-up of 21 months
		Ramalingam 2022 [39]	600	75 (12.5) - who discontinued osimertinib within 60 days of G360 (likely progressors)
	C797X or other mutation	Schoenfeld 2019 [42]	16	1 (6.3)
	G724S mutation	Piotrowska 2022 [37]	35	1 (2.9) - tissue biopsy
	Increased EGFR mutation level	Roper 2020 [58]	6	3 (50.0)
	L718Q/V mutation	Piotrowska 2022 [37]	35[a]	1 (2.9) - tissue biopsy
		Piotrowska 2022 [37]	32[a]	1 (3.1) - liquid biopsy
	Pocket volume reducing (PVR) mutations	Robichaux 2021 [40]	16,715	3 (0.02)
	T790M loss	Cardona 2022 [18]	147	23 (15.4)
	T790M mutation	Ramalingam 2018 [54]	19	0 (0.0)
		Soria 2018 [8]	91	0 (0.0)
**EGFR-independent mechanisms**				
ALK	Fusion	Piotrowska 2022 [37]	32	1 (2.9) - liquid biopsy
BRAF	Fusion	Cardona 2022 [18]	147	2 (1.4)
		Piotrowska 2022 [37]	35	1 (2.9) - tissue biopsy
	Mutation	Cardona 2022 [18]	147	5 (3.4)
	V600E mutation	Piotrowska 2022 [37]	35[a]	1 (2.9) - tissue biopsy
			32[a]	2 (6.0) - liquid biopsy
CCNE1	Amplification	Ramalingam 2022 [39]	1,337	106 (7.9)
CTNNB1	Mutation	Cardona 2022 [18]	147	2 (1.4)
FGF	Amplification	Cardona 2022 [18]	147	1 (0.6)
HER2	Amplification	Cardona 2022 [18]	147	9 (6.2)

Gene name	Resistance mechanism	Author, year	Sample size	N (%) with acquired resistance
	Exon 20 insertion	Ramalingam 2018 [54]	19	1 (5.3)
JAK	Mutation	Cardona 2022 [18]	147	7 (4.8)
JAK2	V617F mutation	Ramalingam 2018 [54]	19	1 (5.3)
KRAS	Mutation	Cardona 2022 [18]	147	7 (4.8)
		Ramalingam 2018 [54]	19	1 (5.3)
MEK1	Mutation	Ramalingam 2018 [54]	19	1 (5.3)
MET	Amplification	Cardona 2022 [18]	147	1 (0.6)
		Piotrowska 2022 [37]	35[a]	7 (20.0) - tissue biopsy
			32[a]	4 (13.0) - liquid biopsy
		Ramalingam 2018 [54]	19	1 (5.3)
		Ramalingam 2022 [39]	1,337	86 (6.4)
		Roper 2020 [58]	9	6 (66.0)
		Soria 2018 [8]	91	14 (15.0)
	Mutation	Cardona 2022 [18]	147	4 (2.7)
PIK3CA	Mutation	Cardona 2022 [18]	147	5 (3.4)
		Ramalingam 2018 [54]	19	1 (5.3)
PTEN	Loss	Cardona 2022 [18]	147	1 (0.6)
RET	Fusion	Cardona 2022 [18]	147	4 (2.7)
TP53	A24fs loss, Y124H loss, Y124C loss	Roper 2020 [58]	9	3 (33.3)
	Mutation	Cardona 2022 [18]	147	43 (29.2)
ALK, RET, BRAF	Fusion	Schoenfeld 2019 [42]	16	0 (0.0)
BRAF, KRAS, HER2	Mutation	Schoenfeld 2019 [42]	16	1 (6.3)
CBL, KRAS, MET, other	Amplification	Roper 2020 [58]	15	1 (6.7)
CD274 (PD-L1), MET	Amplification	Roper 2020 [58]	15	1 (6.7)
CTNNB1, TP53	CTNNB1 S33C mutation, TP53 G112V mutation	Roper 2020 [58]	9	1 (11.1)
HER2, PIK3CA, RAS	HER2 amplification, PIK3CA, RAS mutations	Soria 2018 [8]	91	NA (2.0 - 7.0)

Gene name	Resistance mechanism	Author, year	Sample size	N (%) with acquired resistance
MET, squamous cell	MET Amplification, Squamous cell transformation	Piotrowska 2022 [37]	35	1 (2.9) - tissue biopsy
**EGFR-dependent and EGFR-independent mechanisms**				
EGFR (ErbB1/HER1), HER2 (ErbB2)	Alterations	Cardona 2022 [18]	147	60 (40.8)
EGFR, KRAS	Amplification	Ramalingam 2018 [54]	19	1 (5.3)
EGFR, KRAS, MET	Amplification	Roper 2020 [58]	15	1 (6.7)
EGFR, MET	Amplification	Roper 2020 [58]	15	1 (6.7)
EGFR, HER2, MET	Amplification	Schoenfeld 2019 [42]	16	2 (12.5)
EGFR, MEK1	EGFR G719S mutation, MEK1 G128V mutation	Ramalingam 2018 [54]	19	1 (5.3)
EGFR, MET, TP53	EGFR amplification, MET amplification, TP53 A24fs loss	Roper 2020 [58]	9	1 (11.1)
EGFR, ALK, BRAF	EGFR C797S mutation, EML4-ALK fusion, BRAF V600E mutation	Piotrowska 2022 [37]	32	1 (3.1) - liquid biopsy
EGFR, ESR1	EGFR C797S mutation, ESR1-AKAP12 fusion	Roper 2020 [58]	9	1 (11.1)
**Other mechanisms as reported**				
NA	Mixed NSCLC and SCLC transformation	Roper 2020 [58]	9	1 (11.1)
	Small cell transformation	Cardona 2022 [18]	147	6 (4.1)
		Piotrowska 2022 [37]	35	5 (14.0) - tissue biopsy
		Schoenfeld 2019 [42]	16	1 (6.3)
	Small cell transformation + Squamous cell transformation	Piotrowska 2022 [37]	35	1 (2.9) - tissue biopsy
	Squamous cell transformation	Piotrowska 2022 [37]	35	2 (6.0) - tissue biopsy
	Squamous transformation	Schoenfeld 2019 [42]	16	2 (12.5)

[a] 54 patients had tissue (n = 35) and/or liquid (n = 32) biopsy upon progression on first-line osimertinib

Supplementary Table S5. Resistance mutation profile for patients who received second-line osimertinib

Gene name	Resistance mechanism	Author year	Sample size	N (%) with acquired resistance
**EGFR-dependent mechanisms**				
EGFR	C797G mutation	Papadimitrakopoulou 2018 [59]	73	1 (1.4) - secondary C797 mutation
	C797S mutation	Oxnard 2018 [33]	110	24 (22.0)
		Papadimitrakopoulou 2018 [59]	73	10 (13.7) - secondary C797 mutation
	C797X mutation	Ramalingam 2022 [39]	713	125 (17.5) - 5 years after osimertinib initiation
			713	99 (13.9) – at median follow-up of 26 months
	EGFR sensitizing mutation loss	Hochmair 2018 [24]	39	4 (10.3)
	T790M loss	Oxnard 2018 [33]	110	52 (47.0)
		Hochmair 2018 [24]	39	8 (20.5)
		Papadimitrakopoulou 2018 [59]	73	36 (49.0)
	EGFR amplification, EGFR C797S mutation	Roper 2020 [58]	6	1 (16.7)
**EGFR-independent mechanisms**				
BRAF	BRAF-AGK fusion, BRAF-MKRN1 fusion	Roper 2020 [58]	6	1 (16.7)
	V600E mutation	Papadimitrakopoulou 2018 [59]	73	3 (4.0)
CCNE1	Amplification	Ramalingam 2022 [39]	713	73 (10.3)
HER2	Amplification	Papadimitrakopoulou 2018 [59]	73	4 (5.0)
KRAS	Mutation	Papadimitrakopoulou 2018 [59]	73	1 (1.0)
MET	Amplification	Oxnard 2018 [33]	52	4 (7.7)
		Papadimitrakopoulou 2018 [59]	73	14 (19.0)
		Ramalingam 2022 [39]	713	51 (7.2)
PIK3CA	Amplification	Papadimitrakopoulou 2018 [59]	73	3 (4.0)
	E545K mutation	Papadimitrakopoulou 2018 [59]	73	1 (1.0)
FGFR3, RET, NTRK	Fusion	Papadimitrakopoulou 2018 [59]	73	3 (4.0)

Gene name	Resistance mechanism	Author year	Sample size	N (%) with acquired resistance
**EGFR-dependent and EGFR-independent mechanisms**				
EGFR, BRAF	EGFR T790M mutation, BRAF fusion	Oxnard 2018 [33]	52	1 (1.9)
EGFR, YES1	Amplification	Roper 2020 [58]	6	1 (16.7)
EGFR, MET, other	EGFR C797S mutation, MET amplification, other amplification	Roper 2020 [58]	6	1 (16.7)
**Other mechanisms as reported**				
NA	SCLC transformation	Roper 2020 [58]	6	1 (16.7)
	Unknown by exome, neuroendocrine (NE) differentiation without histologic transformation by RNA-sequencing	Roper 2020 [58]	6	1 (16.7)

Supplementary Table S6. Resistance mutation profile for patients who received first-line osimertinib and beyond

Gene name	Resistance mechanism	Author, year	LoT	Sample size	N (%) with acquired resistance
**EGFR-dependent mechanisms**					
EGFR	Amplification	Bauml 2021-a [17]	1L+	162	8 (4.9)
	A289T mutation	Patil 2019 [34]	1L+	40	2 (5.0)
	C620W mutation	Le 2018 [27]	1L+	19	1 (5.3) - T790M-preserved subgroup
	C797G mutation	Le 2018 [27]	1L+	42	1 (2.4) - total population
			1L+	19	1 (5.3) - T790M-preserved subgroup
	C797S mutation	Bauml 2021-a [17]	1L+	162	18 (11.1)
		Patil 2019 [34]	1L+	40	9 (23.0)
		Le 2018 [27]	1L+	42	8 (19.0) - total population
			1L+	19	8 (42.1) - T790M-preserved subgroup
		Piotrowska 2018 [36]	1L+	22	7 (32.0) - ctDNA analysis
			1L+	3	2 (66.7) - Two distinct metastatic foci biopsied
			1L+	32	6 (19.0) - Tissue biopsy
	C797X or other mutation	Schoenfeld 2019 [42]	1L+	71	11 (15.5)
	G724S mutation	Bauml 2021-a [17]	1L+	162	3 (1.9)

Gene name	Resistance mechanism	Author, year	LoT	Sample size	N (%) with acquired resistance
		Le 2018 [27]	1L+	21	1 (4.8) - T790M-loss subgroup
	L718Q mutation	Patil 2019 [34]	1L+	40	1 (3.0)
		Le 2018 [27]	1L+	21	1 (4.8) - T790M-loss subgroup
	L718Q/V mutation	Bauml 2021-a [17]	1L+	162	2 (1.2)
	L792 H/V mutation	Patil 2019 [34]	1L+	40	2 (5.0)
	L792H mutation	Le 2018 [27]	1L+	42	2 (4.8) - total population
			1L+	19	2 (10.5) - T790M-preserved subgroup
	T790M loss	Bauml 2021-a [17]	1L+	162	64 (39.5)
		Schoenfeld 2019 [42]	1L+	71	17 (23.9)
		Le 2018 [27]	1L+	42	21 (50.0)
		Patil 2019 [34]	1L+	40	19 (48.0)
	P596L mutation	Le 2018 [27]	1L+	21	1 (4.8) - T790M-loss subgroup
	V834L mutation	Le 2018 [27]	1L+	21	1 (4.8) - T790M-loss subgroup
**EGFR-independent mechanisms**					
ALK	EML4-ALK fusion	Patil 2019 [34]	1L+	40	1 (3.0)
BRAF	V600E mutation	Patil 2019 [34]	1L+	40	1 (3.0)
	Mutation, amplification	Bauml 2021-a [17]	1L+	162	5 (3.1)
BRCA 1/2	Mutation	Patil 2019 [34]	1L+	40	3 (8.0)
CDKN2A/B	Mutation	Bauml 2021-a [17]	1L+	162	10 (6.2)
ERBB2	Amplification	Le 2018 [27]	1L+	21	1 (4.8) - T790M-loss subgroup
FGFR 1/2	Mutation	Patil 2019 [34]	1L+	40	4 (10.0)
FGFR1	Mutation	Bauml 2021-a [17]	1L+	162	3 (1.9)
FGFR2	Mutation	Bauml 2021-a [17]	1L+	162	0 (0)
FGFR3	Mutation	Bauml 2021-a [17]	1L+	162	2 (1.2)
FGFR3/FGFR19	FGFR3/FGFR19 amplification	Le 2018 [27]	1L+	21	1 (4.8) - T790M-loss subgroup
FGFR4	Mutation	Bauml 2021-a [17]	1L+	162	1 (0.6)
GNAS	Mutation	Patil 2019 [34]	1L+	40	3 (8.0)
KRAS	Amplification	Bauml 2021-a [17]	1L+	162	4 (2.5)
	Mutation	Bauml 2021-a [17]	1L+	162	12 (7.4)

Gene name	Resistance mechanism	Author, year	LoT	Sample size	N (%) with acquired resistance
		Patil 2019 [34]	1L+	40	2 (5.0)
	KRAS Q61R mutation	Le 2018 [27]	1L+	21	1 (4.8) - T790M-loss subgroup
MET	Amplification	Bauml 2021-a [17]	1L+	162	18 (11.1)
		Patil 2019 [34]	1L+	22	2 (10.0) - tissue biopsy
		Piotrowska 2018 [36]	1L+	32	7 (22.0) - tissue biopsy
			1L+	22	5 (23.0) - ctDNA analysis
			1L+	3	1 (33.3) - two distinct metastatic foci biopsied
		Le 2018 [27]	1L+	42	6 (14.3) - total population
			1L+	19	5 (26.3) - T790M-preserved subgroup
			1L+	21	1 (4.8) - T790M-loss subgroup
	Mutation	Bauml 2021-a [17]	1L+	162	3 (1.9)
Non-BRAF	V600E mutation	Patil 2019 [34]	1L+	40	4 (10.0)
PDFGRA	Mutation	Patil 2019 [34]	1L+	40	2 (5.0)
PIK3CA	Amplification	Bauml 2021-a [17]	1L+	162	0 (0)
	Mutation	Bauml 2021-a [17]	1L+	162	15 (9.3)
		Patil 2019 [34]	1L+	40	2 (5.0)
	PIK3CA E418K/E542K mutation	Le 2018 [27]	1L+	21	1 (4.8) - T790M-loss subgroup
	PIK3CA E542K/E545K mutation	Le 2018 [27]	1L+	21	1 (4.8) - T790M-loss subgroup
ALK, BRAF, FGFR3, NTRK1, RET	Rearrangements or fusions	Bauml 2021-a [17]	1L+	162	5 (3.1)
ALK, RET, BRAF	Fusions	Schoenfeld 2019 [42]	1L+	71	5 (7.0)
KRAS, BRAF, HER2	Mutation	Schoenfeld 2019 [42]	1L+	71	5 (7.0)
**EGFR-dependent and EGFR-independent mechanisms**					
RB1/TP53/PIK3CA	RB1/TP53/PIK3CA alterations	Le 2018 [27]	1L+	21	1 (4.8) - T790M-loss subgroup
CDK4/KRAS/MDM2	CDK4/KRAS/MDM2 amplification	Le 2018 [27]	1L+	21	1 (4.8) - T790M-loss subgroup
EGFR, MET, HER2	Amplification	Schoenfeld 2019 [42]	1L+	71	9 (12.7)

Gene name	Resistance mechanism	Author, year	LoT	Sample size	N (%) with acquired resistance
EGFR, AXL	EGFR T790M preserved, EGFR C797S mutation, AXL mutation	Le 2018 [27]	1L+	8	1 (12.5) - epithelial mesenchymal transition (EMT) associated mechanism
Not reported	Any acquired osimertinib-resistance mechanism	Roper 2020 [58]	1L, 2L	15	14 (93.0)
	Two or more co-existing mechanisms		1L, 2L	15	11 (73.0)
**Other mechanisms as reported**					
NA	Small cell transformation	Piotrowska 2018 [36]	1L+	32	2 (6.3) - Tissue biopsy
		Schoenfeld 2019 [42]	1L+	71	5 (7.0)
	Squamous cell transformation	Piotrowska 2018 [36]	1L+	32	1 (3.1) - Tissue biopsy
		Schoenfeld 2019 [42]	1L+	71	5 (7.0)

Supplementary Table S7 Resistance mutation profile for patients who received second-line osimertinib and beyond

Gene name	Resistance mechanism	Author, year	LoT	Sample size	N (%) with acquired resistance
**EGFR-dependent mechanisms**					
EGFR	Activating mutations and T790M loss	Zhao 2018 [50]	2L+	293	66 (22.5)
	C797S mutation	Lim 2021 [29]	2L+	33	4 (11.0)
		Zhao 2018 [50]	2L+	293	60 (20.5)
		Oxnard 2018 [33]	2L, 3L	41	9 (22.0) - overall population
		Oxnard 2018 [33]	2L, 3L	13	9 (69.0) - T790M-maintained subgroup
		Oxnard 2018 [33]	2L, 3L	28	0 (0) - T790M-loss subgroup
	C797X or other mutation	Schoenfeld 2019 [42]	2L+	55	10 (18.2)
	G724S mutation	Oxnard 2018 [33]	2L, 3L	41	1 (2.4)
	G824D mutation	Lim 2021 [29]	2L+	33	2 (6.0)
	T790M loss	Schoenfeld 2019 [42]	2L+	55	17 (30.9)
		Zhao 2018 [50]	2L+	293	130 (44.4)
		Oxnard 2018 [33]	2L, 3L	41	28 (68.3)

Gene name	Resistance mechanism	Author, year	LoT	Sample size	N (%) with acquired resistance
	V726M mutation	Lim 2021 [29]	2L+	33	1 (3.0)
	V843I mutation	Lim 2021 [29]	2L+	33	1 (3.0)
	L792H/V mutation, G796S mutation, L718Q/V mutation, G719A mutation, or E709K mutation	Zhao 2018 [50]	2L+	293	27 (9.2)
**EGFR-independent mechanisms**					
BRAF	Activating mutation	Zhao 2018 [50]	2L+	293	8 (2.7)
	Mutation	Oxnard 2018 [33]	2L, 3L	28	2 (7.1) - T790M-loss subgroup
ERbB2	Activating mutation/amplification	Zhao 2018 [50]	2L+	293	14 (4.8)
ESYT2-BRAF	Fusion	Oxnard 2018 [33]	2L, 3L	28	1 (3.6) - T790M-loss subgroup
FGFR3-TACC3	Fusion	Oxnard 2018 [33]	2L, 3L	28	1 (3.6) - T790M-loss subgroup
HER2	Amplification	Lim 2021 [29]	2L+	33	2 (6.0)
KRAS	Activating mutation	Zhao 2018 [50]	2L+	293	5 (1.7)
	G12V mutation	Oxnard 2018 [33]	2L, 3L	41	1 (2.4)
	Q61K mutation	Oxnard 2018 [33]	2L, 3L	28	1 (3.6) - T790M-loss subgroup
MET	Amplification	Zhao 2018 [50]	2L+	293	16 (5.5)
		Lim 2021 [29]	2L+	33	3 (9.0)
		Oxnard 2018 [33]	2L, 3L	41	4 (9.8) – overall population
		Oxnard 2018 [33]	2L, 3L	28	4 (14.3) - T790M-loss subgroup
NR	Non-EGFR, non-MET mechanisms	Bauml 2021-b [55]	2L, 3L	45	10 (22.2)
PIK3CA	PI3K-AKT-mTOR signaling activating mutations	Zhao 2018 [50]	2L+	293	37 (12.6)
	Mutation	Oxnard 2018 [33]	2L, 3L	41	4 (9.8) – overall population
				13	2 (15.4) - T790M-maintained subgroup
				28	2 (7.1) - T790M-loss subgroup
Rb1	Loss of function mutation	Zhao 2018 [50]	2L+	293	24 (8.2)
RET	Fusion	Oxnard 2018 [33]	2L, 3L	28	1 (3.6) - T790M-loss subgroup
TP53	Mutation or 2-copy loss	Oxnard 2018 [33]	2L, 3L	41	26 (63.4) - detected in resistance biopsies, but not specified as acquired after treatment

Gene name	Resistance mechanism	Author, year	LoT	Sample size	N (%) with acquired resistance
ALK or ROS1 or RET	ALK or ROS1 or RET fusion	Zhao 2018 [50]	2L+	293	9 (3.1)
ALK, RET, BRAF	Fusion	Schoenfeld 2019 [42]	2L+	55	5 (9.1)
KRAS, BRAF, HER2	Mutation	Schoenfeld 2019 [42]	2L+	55	4 (7.3)
**EGFR-dependent and EGFR-independent mechanisms**					
EGFR, HER2, MET	Amplification	Schoenfeld 2019 [42]	2L+	55	7 (12.7)
EGFR, NR	Multiple	Zhao 2018 [50]	2L+	293	31 (10.6)
EGFR, MET, ALK	EGFR T790M mutation, MET amplification, ALK re-arrangement	Oxnard 2018 [33]	2L, 3L	41	1 (2.4)
EGFR, MET	Mutation or amplification	Bauml 2021-b [55]	2L, 3L	45	17 (37.8)
EGFR and others	Known resistance mechanisms (unspecified)	Zhao 2018 [50]	2L+	66	11 (16.7) – EGFR activating mutation and T790M-loss subgroup
			2L+	130	56 (43.1) - T790M-loss subgroup
			2L+	97	62 (63.9) - EGFR activating mutation and T790M-maintained subgroup
**Other mechanisms as reported**					
NA	Small cell transformation	Schoenfeld 2019 [42]	2L+	55	4 (7.3)
	Squamous transformation	Schoenfeld 2019 [42]	2L+	55	3 (5.5)
	Small-cell lung cancer transformation	Oxnard 2018 [33]	2L, 3L	28	6 (21.4) - T790M-loss subgroup
NR	Unknown mechanisms	Bauml 2021-b [55]	2L, 3L	45	18 (40)

Supplementary Table S8. Resistance mutation profile for patients who received TKIs or non-TKIs where osimertinib was one of the treatment options

Gene name	Resistance mechanism	Author, year	LoT	Treatment name	Sample size	N (%) with acquired resistance
**EGFR-dependent mechanisms**						
EGFR	T790M mutation	Mondaca 2019 [32]	1L	Erlotinib, Afatinib, Gefitinib, Osimertinib, Rociletinib, Nazartinib	177	56 (32.0)
		Patil 2020 [35]	Unspecified	Erlotinib, Afatinib, Osimertinib	36	26 (72.0) - Exon 19del subgroup
					20	13 (65.0) - L858R subgroup
				Gefitinib, Erlotinib, Afatinib, Osimertinib	6	2 (33.0) - G719X subgroup
				Erlotinib, Osimertinib	2	0 (0.0) - Exon 20ins subgroup
	C797G mutation	Goldberg 2018 [21]	Unspecified	Osimertinib, Rociletinib	29	2 (6.9)
	C797S mutation	Goldberg 2018 [21]	Unspecified	Osimertinib, Rociletinib	29	23 (79.3)
	C797S alteration	Mack 2020 [30]	Unspecified	Osimertinib, Rociletinib, Other	447	5 (1.1)
	C797X mutation	Markovets 2021 [57]	1L+	Osimertinib + Savolitinib	45	7 (16.0)
	L718Q alteration	Mack 2020 [30]	Unspecified	Erlotinib, Afatinib, Gefitinib, Osimertinib, Rociletinib, Other	447	1 (0.2)
	L792H mutation	Goldberg 2018 [21]	Unspecified	Osimertinib, Rociletinib	29	1 (3.4)
	T790M alteration	Mack 2020 [30]	Unspecified	Erlotinib, Afatinib, Gefitinib, Osimertinib, Rociletinib, Other	447	223 (50.0)
	T854A alteration	Mack 2020 [30]	Unspecified	Erlotinib, Afatinib, Gefitinib, Osimertinib, Rociletinib, Other	447	1 (0.2)
	C797S mutation, C797N mutation	Goldberg 2018 [21]	Unspecified	Osimertinib, Rociletinib	29	1 (3.4)
	C797S mutation, L792H mutation	Goldberg 2018 [21]	Unspecified	Osimertinib, Rociletinib	29	2 (6.9)
**EGFR-independent mechanisms**						
ALK	Fusion	Mack 2020 [30]	Unspecified	Erlotinib, Afatinib, Gefitinib, Osimertinib, Rociletinib, Other	447	1 (0.2)
					65	32 (49.0) - ALK fusion positive subgroup
					65	5 (7.7) - ALK fusion positive subgroup

Gene name	Resistance mechanism	Author, year	LoT	Treatment name	Sample size	N (%) with acquired resistance
	EML4 fusion	Schrock 2018 [43]	Unspecified	Erlotinib, Osimertinib or Afatinib or Erlotinib, Afatinib	12	3 (25.0) - With pre and post treatment NGS samples.
	STRN fusion	Schrock 2018 [43]	Unspecified	Erlotinib, Osimertinib	14	1 (7.1) - With acquired kinase fusions
BRAF	Amplification	Mambetsariev 2022 [31]	1L+	Erlotinib, Osimertinib, Afatinib, Carboplatin/Pemetrexed, Carboplatin/Pemetrexed/Pembrolizumab	9	1 (11.1)
	Re-arrangement	Mambetsariev 2022 [31]	1L+	Erlotinib, Osimertinib, Afatinib, Carboplatin/Pemetrexed, Carboplatin/Pemetrexed/Pembrolizumab	9	1 (11.1)
	Fusion	Mambetsariev 2022 [31]	1L+	Erlotinib, Osimertinib, Afatinib, Carboplatin/Pemetrexed, Carboplatin/Pemetrexed/Pembrolizumab	9	1 (11.1)
	AGK fusion	Piotrowska 2018 [36]	1L, 2L	Erlotinib, Osimertinib	24	1 (4.2) - Tissue testing from biopsies of progressing lesions
		Schrock 2018 [43]	Unspecified	Erlotinib, Osimertinib	12	1 (8.3) - With pre and post treatment NGS samples.
	PCBP2 fusion	Piotrowska 2018 [36]	1L - 3L	Erlotinib, Carboplatin/Pemetrexed, Osimertinib	24	1 (4.2) - Tissue testing from biopsies of progressing lesions
	BAIAP2L1 fusion	Piotrowska 2018 [36]	1L - 4L	Erlotinib, Osimertinib, Carboplatin/Pemetrexed, Osimertinib/Gemcitabine	3	1 (33.3) - EGFR mutant subset of tissue testing
	SNV alteration	Mack 2020 [30]	Unspecified	Erlotinib, Afatinib, Gefitinib, Osimertinib, Rociletinib, Other	447	7 (1.6)
	G469A/G469R activating mutation	Mack 2020 [30]	Unspecified	Erlotinib, Afatinib, Gefitinib, Osimertinib, Rociletinib, Other	447	3 (0.7)
	L485F activating mutation	Mack 2020 [30]	Unspecified	Erlotinib, Afatinib, Gefitinib, Osimertinib, Rociletinib, Other	447	1 (0.2)
	V600E activating mutation	Mack 2020 [30]	Unspecified	Erlotinib, Afatinib, Gefitinib, Osimertinib, Rociletinib, Other	447	3 (0.7)
ERBB2	Amplification	Mack 2020 [30]	Unspecified	Erlotinib, Afatinib, Gefitinib, Osimertinib, Rociletinib, Other	447	24 (5.4)
FGFR3	Fusion	Mack 2020 [30]	Unspecified	Erlotinib, Afatinib, Gefitinib, Osimertinib, Rociletinib, Other	447	3 (0.7)

Gene name	Resistance mechanism	Author, year	LoT	Treatment name	Sample size	N (%) with acquired resistance
	TACC3 fusion	Schrock 2018 [43]	Unspecified	Erlotinib, ASP8273 or Afatinib + Cetuximab or Afatinib or Erlotinib, Osimertinib	12	4 (33.3) - With pre and post treatment NGS samples.
KIT	Mutation	Yu 2022 [48]	Unspecified	Gefitinib, Afatinib, Erlotinib, or Osimertinib	5	1 (20.0)
KRAS	G12X mutation, G13X mutation	Markovets 2021 [57]	1L+	Osimertinib + Savolitinib	45	5 (11.0)
	SNV alteration	Mack 2020 [30]	Unspecified	Erlotinib, Afatinib, Gefitinib, Osimertinib, Rociletinib, Other	447	6 (1.3)
MET	Amplification	Mack 2020 [30]	Unspecified	Erlotinib, Afatinib, Gefitinib, Osimertinib, Rociletinib, Other	447	51 (11.4)
		Patil 2020 [35]	Unspecified	Erlotinib, Afatinib, Osimertinib	36	4 (11.0) - Exon 19del subgroup
					20	1 (5.0) - L858R subgroup
				Gefitinib, Erlotinib, Afatinib, Osimertinib	6	0 (0) - G719X subgroup
				Erlotinib, Osimertinib	1	0 (0) - Exon 20ins subgroup
	D1228X mutation, Y1230X mutation, L1212X mutation	Markovets 2021 [57]	1L+	Osimertinib + Savolitinib	45	9 (20.0)
	Multiple mutations	Markovets 2021 [57]	1L+	Osimertinib + Savolitinib	9	7 (77.8) - MET positive subgroup
NE1	Loss	Mack 2020 [30]	Unspecified	Erlotinib, Afatinib, Gefitinib, Osimertinib, Rociletinib, Other	447	11 (2.5)
NTRK1	Fusion	Mack 2020 [30]	Unspecified	Erlotinib, Afatinib, Gefitinib, Osimertinib, Rociletinib, Other	447	1 (0.2)
PIK3CA	Alteration	Mambetsariev 2022 [31]	1L+	Erlotinib, Osimertinib, Afatinib, Carboplatin/Pemetrexed, Carboplatin/Pemetrexed/Pembrolizumab	9	4 (44.4)
	E545K mutation	Markovets 2021 [57]	1L+	Osimertinib + Savolitinib	45	2 (4.0)
	SNV alteration	Mack 2020 [30]	Unspecified	Erlotinib, Afatinib, Gefitinib, Osimertinib, Rociletinib, Other	447	40 (8.9)
RB1	Alteration	Mambetsariev 2022 [31]	1L+	Erlotinib, Osimertinib, Afatinib, Carboplatin/Pemetrexed, Carboplatin/Pemetrexed/Pembrolizumab	9	3 (33.3)

Gene name	Resistance mechanism	Author, year	LoT	Treatment name	Sample size	N (%) with acquired resistance
	Loss	Mack 2020 [30]	Unspecified	Erlotinib, Afatinib, Gefitinib, Osimertinib, Rociletinib, Other	447	11 (2.5)
		Yu 2022 [48]	Unspecified	Gefitinib, Afatinib, Erlotinib, or Osimertinib	5	1 (20.0)
					2	1 (50.0) - NGS after transformation only
RET	Fusion	Mack 2020 [30]	Unspecified	Erlotinib, Afatinib, Gefitinib, Osimertinib, Rociletinib, Other	447	2 (0.4)
	TRIM24 fusion	Schrock 2018 [43]	Unspecified	Erlotinib, Osimertinib	14	1 (7.1) - With acquired kinase fusions
ALK, BRAF	ALK fusion, BRAF V600E mutation	Schrock 2018 [43]	Unspecified	Erlotinib, Osimertinib	31	2 (6.4)
BRAF, CCNE1	BRAF substitution, CCNE1 amplification	Mambetsariev 2022 [31]	1L+	Erlotinib, Osimertinib, Afatinib, Carboplatin/Pemetrexed, Carboplatin/Pemetrexed/Pembrolizumab	9	1 (11.1)
GNAS, TP53, BRAF	GNAS mutation, TP53 loss, BRAF amplification	Mambetsariev 2022 [31]	1L+	Erlotinib, Osimertinib, Afatinib, Carboplatin/Pemetrexed, Carboplatin/Pemetrexed/Pembrolizumab	9	1 (11.1)
PPARG, CD22, BCL6	Mutation	Yu 2022 [48]	Unspecified	Gefitinib, Afatinib, Erlotinib, or Osimertinib	5	1 (20.0)
RET, NTRK1	RET CCDC6 fusion, NTRK1 TPM3 fusion	Piotrowska 2018 [36]	1L, 2L	Erlotinib, Osimertinib	1	1 (100.0)
RTK or BRAF	Fusion	Schrock 2018 [43]	Unspecified	Erlotinib then Osimertinib	12	1 (8.3) - With pre and post treatment NGS samples.
				Erlotinib, Afatinib, Osimertinib	12	1 (8.3) - With pre and post treatment NGS samples.
RPTOR, ABL2, IRS2, KLHL6, PDGFRB, GNAS, AURKA, PTCH1, SMAD2, FLT3, KDM5A	Mutation	Yu 2022 [48]	Unspecified	Gefitinib, Afatinib, Erlotinib, or Osimertinib	5	1 (20.0)
TP53, PIK3CA, RB1, POLE, MRE11A	Alteration	Mambetsariev 2022 [31]	1L+	Erlotinib, Osimertinib, Afatinib, Carboplatin/Pemetrexed, Carboplatin/Pemetrexed/Pembrolizumab	9	1 (11.1)

Gene name	Resistance mechanism	Author, year	LoT	Treatment name	Sample size	N (%) with acquired resistance
	Substitution	Mambetsariev 2022 [31]	1L+	Erlotinib, Osimertinib, Afatinib, Carboplatin/Pemetrexed, Carboplatin/Pemetrexed/Pembrolizumab	9	1 (11.1)
ZRSR2, FOXO1, KDR, SMAD4, RANBP2	Mutation	Yu 2022 [48]	Unspecified	Gefitinib, Afatinib, Erlotinib, or Osimertinib	5	1 (20.0)
**EGFR-dependent and EGFR-independent mechanisms**						
EGFR, TP53	Mutation	Mambetsariev 2022 [31]	1L+	Erlotinib, Osimertinib, Afatinib, Carboplatin/Pemetrexed, Carboplatin/Pemetrexed/Pembrolizumab	9	1 (11.1)
WNK1, APC, KMT2C, ATRX, PIAS4, SRP68, TERT, LMNA, CBLB, RNF139, PTPRT, MALT1, CFTR, CRLF2, AMER1, TOP2A, SLC47A2, BCLAF1, ZMYM3, HSP90AA1, EGFR amplification	Mutation	Yu 2022 [48]	Unspecified	Gefitinib, Afatinib, Erlotinib, or Osimertinib	5	1 (20.0)
EGFR, RTK	EGFR-RTK FGFR1 fusion	Schrock 2018 [43]	Unspecified	Erlotinib, Osimertinib	14	1 (7.1) - With acquired kinase fusions
EGFR, RET	EGFR T790M mutation, RET CCDC6 fusion	Piotrowska 2018 [36]	1L, 2L	Afatinib, Osimertinib	24	1 (4.2) - Tissue testing from biopsies of progressing lesions
	Second-site EGFR C797S mutation, RET G810S mutation	Rotow 2021 [41]	2L, 2L+	Osimertinib + Selpercatinib	10	1 (10.0)
	EGFR T790M mutation, EGFR C797S mutation, RET CCDC6 fusion	Yao 2019 [47]	Unspecified	Osimertinib	7	1 (14.3%) - among cases with RET fusion
EGFR, ALK	EGFR T790M mutation, ALK PLEKHA7 fusion	Schrock 2018 [43]	Unspecified	Erlotinib, Afatinib, Osimertinib	12	1 (8.3) - With pre and post treatment NGS samples
**Other mechanisms as reported**						

Gene name	Resistance mechanism	Author, year	LoT	Treatment name	Sample size	N (%) with acquired resistance
NA	Small cell transformation	Mambetsariev 2022 [31]	1L+	Erlotinib, Osimertinib, Afatinib, Carboplatin/Pemetrexed, Carboplatin/Pemetrexed/Pembrolizumab	9	9 (100.0)

Supplementary Table S9 Resistance mutation profile for patients who received other TKIs or non-TKIs

Gene name	Resistance mechanism	Author, year	LoT	Treatment name	Sample size	N (%) with acquired resistance
**EGFR-dependent mechanisms**						
EGFR	Amplification	Elamin 2022 [51]	1L+	Poziotinib	14	1 (7.1) - D770_N771dupDN resistance
					14	1 (7.1) - H773dupH resistance
					20	2 (10.0) - Responding patients
	T790M mutation	Soria 2018 [8]	1L	Gefitinib or Erlotinib	129	60 (47.0)
		McCoach 2021 [53]	1L+	Capmatinib + Erlotinib	12	1 (8.3)
		Elamin 2022 [51]	1L+	Poziotinib	23	3 (13.0)
		Elamin 2022 [51]	1L+	Poziotinib	4	2 (50.0) - Responding patients with EGFR point mutations
		Patil 2020 [35]	Unspecified	Erlotinib, Gefitinib, Afatinib	65	59 (90.8)
				Erlotinib	1	0 (0.0)
	C797S mutation	Lu 2021 [52]	1L+	Aumolertinib	42	7 (16.7)
		Helman 2018 [23]	Unspecified	Rociletinib	65	3 (4.6) - ctDNA positive plasma
	D770A mutation	Elamin 2022 [51]	1L+	Poziotinib	4	1 (25.0) - Responding patients with EGFR point mutations
	D770A mutation, V1128I mutation	Elamin 2022 [51]	1L+	Poziotinib	14	1 (7.1) - A767_V769dupASV resistance
	L718Q mutation	Lu 2021 [52]	1L+	Aumolertinib	42	1 (2.4)
	T790M, V774A, D770A point mutations	Elamin 2022 [51]	1L+	Poziotinib	20	4 (20.0) - Responding patients

Gene name	Resistance mechanism	Author, year	LoT	Treatment name	Sample size	N (%) with acquired resistance
	V774A mutation	Elamin 2022 [51]	1L+	Poziotinib	4	1 (25.0) - Responding patients with EGFR point mutations
	NR	Elamin 2022 [51]	1L+	Poziotinib	23	4 (17.4)
	Alteration	Helman 2018 [23]	Unspecified	Rociletinib	58	21 (36.0) - Co-mutation variants
**EGFR-independent mechanisms**						
ALK	Alteration	Helman 2018 [23]	Unspecified	Rociletinib	58	2 (3.0) - Co-mutation variants
	EML4 fusion	Schrock 2018 [43]	Unspecified	Afatinib	14	1 (7.1) - With Acquired RTK or BRAF Fusions
APC	Alteration	Helman 2018 [23]	Unspecified	Rociletinib	58	4 (7.0) - Co-mutation variants
AR	Alteration	Helman 2018 [23]	Unspecified	Rociletinib	58	1 (2.0) - Co-mutation variants
ARID1A	Alteration	Helman 2018 [23]	Unspecified	Rociletinib	58	4 (7.0) - Co-mutation variants
ATM	Alteration	Helman 2018 [23]	Unspecified	Rociletinib	58	1 (2.0) - Co-mutation variants
BRAF	Alteration	Helman 2018 [23]	Unspecified	Rociletinib	58	2 (3.0) - Co-mutation variants
	DOCK4 fusion	Schrock 2018 [43]	Unspecified	Erlotinib	14	1 (7.1) - With Acquired RTK or BRAF Fusions
	EPS515 fusion	Schrock 2018 [43]	Unspecified	Erlotinib	14	1 (7.1) - With Acquired RTK or BRAF Fusions
	SALL2 fusion	Schrock 2018 [43]	Unspecified	Erlotinib	14	1 (7.1) - With Acquired RTK or BRAF Fusions
	Mutation	Helman 2018 [23]	Unspecified	Rociletinib	66	1 (2.0) - With mutations in MAPK signaling pathway genes
BRCA1	Alteration	Helman 2018 [23]	Unspecified	Rociletinib	58	1 (2.0) - Co-mutation variants
BRCA2	Alteration	Helman 2018 [23]	Unspecified	Rociletinib	58	3 (5.0) - Co-mutation variants
CCND1	Alteration	Helman 2018 [23]	Unspecified	Rociletinib	58	1 (2.0) - Co-mutation variants
CCND2	Alteration	Helman 2018 [23]	Unspecified	Rociletinib	58	4 (7.0) - Co-mutation variants
CDK4	Alteration	Helman 2018 [23]	Unspecified	Rociletinib	58	1 (2.0) - Co-mutation variants

Gene name	Resistance mechanism	Author, year	LoT	Treatment name	Sample size	N (%) with acquired resistance
CDK6	Amplification	Elamin 2022 [51]	1L+	Poziotinib	14	1 (7.1) - D770_N771insG resistance
					20	2 (10.0) - Responding patients
	Alteration	Helman 2018 [23]	Unspecified	Rociletinib	58	2 (3.0) - Co-mutation variants
CDKN2A	Alteration	Helman 2018 [23]	Unspecified	Rociletinib	58	2 (3.0) - Co-mutation variants
CTNNB1	Alteration	Helman 2018 [23]	Unspecified	Rociletinib	58	1 (2.0) - Co-mutation variants
DDR	R714Q mutation	Elamin 2022 [51]	1L+	Poziotinib	14	2 (14.3) - P772_H773insPNP
ERBB2	Alteration	Helman 2018 [23]	Unspecified	Rociletinib	58	2 (3.0) - Co-mutation variants
ESRI	Alteration	Helman 2018 [23]	Unspecified	Rociletinib	58	1 (2.0) - Co-mutation variants
FGFR1	Alteration	Helman 2018 [23]	Unspecified	Rociletinib	58	3 (5.0) - Co-mutation variants
FGFR2	Alteration	Helman 2018 [23]	Unspecified	Rociletinib	58	2 (3.0) - Co-mutation variants
FGFR3	Alteration	Helman 2018 [23]	Unspecified	Rociletinib	58	1 (2.0) - Co-mutation variants
	TACC3 fusion	Schrock 2018 [43]	Unspecified	Erlotinib	14	1 (7.1) - With Acquired RTK or BRAF Fusions
GNAS	R201C mutation	Elamin 2022 [51]	1L+	Poziotinib	14	1 (7.1) - S768_D770dupSVD resistance
	Alteration	Helman 2018 [23]	Unspecified	Rociletinib	58	3 (5.0) - Co-mutation variants
HER2	Amplification	Soria 2018 [8]	1L	Gefitinib or Erlotinib	129	3 (2.0)
HNF1A	Alteration	Helman 2018 [23]	Unspecified	Rociletinib	58	1 (2.0) - Co-mutation variants
HRAS	Alteration	Helman 2018 [23]	Unspecified	Rociletinib	58	2 (3.0) - Co-mutation variants
	Mutation	Helman 2018 [23]	Unspecified	Rociletinib	66	2 (3.0) - With mutations in MAPK signaling pathway genes
KIT	Alteration	Helman 2018 [23]	Unspecified	Rociletinib	58	2 (3.0) - Co-mutation variants
KRAS	Alteration	Helman 2018 [23]	Unspecified	Rociletinib	58	2 (3.0) - Co-mutation variants

Gene name	Resistance mechanism	Author, year	LoT	Treatment name	Sample size	N (%) with acquired resistance
	Mutation	Helman 2018 [23]	Unspecified	Rociletinib	66	2 (3.0) - With mutations in MAPK signaling pathway genes
MAP2K1	Mutation	Helman 2018 [23]	Unspecified	Rociletinib	66	<1.0% - With mutations in MAPK signaling pathway genes
MAP2K1/3	Mutation	Helman 2018 [23]	Unspecified	Rociletinib	66	<1.0% - With mutations in MAPK signaling pathway genes
MAP2K2	S94L mutation	Elamin 2022 [51]	1L+	Poziotinib	14	1 (7.1) - NAN771dupN resistance
					20	1 (5.0) - Responding patients
	Mutation	Helman 2018 [23]	Unspecified	Rociletinib	66	2 (3.0) - With mutations in MAPK signaling pathway genes
	Alteration	Helman 2018 [23]	Unspecified	Rociletinib	58	2 (3.0) - Co-mutation variants
MET	Amplification	Soria 2018 [8]	1L	Gefitinib or Erlotinib	129	5 (4.0)
		Elamin 2022 [51]	1L+	Poziotinib	20	1 (5.0) - Responding patients
	Amplification	Helman 2018 [23]	Unspecified	Rociletinib	66	5 (7.6) - Plasma collected at time of progression
		Patil 2020 [35]	Unspecified	Erlotinib	1	0 (0.0)
	Focal amplification	Helman 2018 [23]	Unspecified	Rociletinib	65	3 (4.6) - ctDNA positive plasma
					66	3 (4.5) - Plasma collected at time of progression
	S204 deletion	Elamin 2022 [51]	1L+	Poziotinib	14	1 (7.1) - P772_H773dupPH resistance
	Alteration	Helman 2018 [23]	Unspecified	Rociletinib	58	5 (9.0) - Co-mutation variants
MYC	Alteration	Helman 2018 [23]	Unspecified	Rociletinib	58	5 (9.0) - Co-mutation variants
NF1	Alteration	Helman 2018 [23]	Unspecified	Rociletinib	58	6 (10.0) - Co-mutation variants
NOTCH1	Alteration	Helman 2018 [23]	Unspecified	Rociletinib	58	3 (5.0) - Co-mutation variants
NR	NR	Elamin 2022 [51]	1L+	Poziotinib	23	11 (47.8)
NRAS	Alteration	Helman 2018 [23]	Unspecified	Rociletinib	58	1 (2.0) - Co-mutation variants
	Mutation	Helman 2018 [23]	Unspecified	Rociletinib	66	1 (2.0) - With mutations in MAPK signaling pathway genes

Gene name	Resistance mechanism	Author, year	LoT	Treatment name	Sample size	N (%) with acquired resistance
NTRK1	Alteration	Helman 2018 [23]	Unspecified	Rociletinib	58	1 (2.0) - Co-mutation variants
	TPM fusion	Schrock 2018 [43]	Unspecified	Erlotinib	12	1 (8.3) - With pre and post treatment NGS samples.
PDGFRA	N352K mutation	Elamin 2022 [51]	1L+	Poziotinib	14	1 (7.1) - S768_D770dupSVD resistance
	Alteration	Helman 2018 [23]	Unspecified	Rociletinib	58	5 (9.0) - Co-mutation variants
PIK3CA	E545K mutation	Elamin 2022 [51]	1L+	Poziotinib	20	1 (5.0) - Responding patients
		Helman 2018 [23]	Unspecified	Rociletinib	65	3 (4.6) - ctDNA positive plasma
	E545K mutation, H1047R mutation	Elamin 2022 [51]	1L+	Poziotinib	14	1 (7.1) - S768_D770dupSVD resistance
	Alteration	Helman 2018 [23]	Unspecified	Rociletinib	58	10 (17.0) - Co-mutation variants
PTEN	Alteration	Helman 2018 [23]	Unspecified	Rociletinib	58	4 (7.0) - Co-mutation variants
PTPN11	Alteration	Helman 2018 [23]	Unspecified	Rociletinib	58	1 (2.0) - Co-mutation variants
RAF1	Alteration	Helman 2018 [23]	Unspecified	Rociletinib	58	2 (3.0) - Co-mutation variants
	Mutation	Helman 2018 [23]	Unspecified	Rociletinib	66	1 (2.0) - With mutations in MAPK signaling pathway genes
RB1	Alteration	Helman 2018 [23]	Unspecified	Rociletinib	58	2 (3.0) - Co-mutation variants
RET	CCDC6 fusion	Piotrowska 2018 [36]	1L, 2L	Cisplatin/Pemetrexed, Afatinib	3	1 (33.3)
		Schrock 2018 [43]	Unspecified	Erlotinib	12	2 (16.7) - With pre and post treatment NGS samples
					14	1 (7.1) - With Acquired RTK or BRAF Fusions
	NCOA4 fusion	Piotrowska 2018 [36]	1L, 2L	Cisplatin/Pemetrexed, Afatinib/Cetuximab	3	1 (33.3)
		Schrock 2018 [43]	Unspecified	Afatinib	14	1 (7.1) - With Acquired RTK or BRAF Fusions
	Alteration	Helman 2018 [23]	Unspecified	Rociletinib	58	2 (3.0) - Co-mutation variants

Gene name	Resistance mechanism	Author, year	LoT	Treatment name	Sample size	N (%) with acquired resistance
RIT1	Mutation	Helman 2018 [23]	Unspecified	Rociletinib	66	<1.0%- With mutations in MAPK signaling pathway genes
SMAD4	Alteration	Helman 2018 [23]	Unspecified	Rociletinib	58	3 (5.0) - Co-mutation variants
TERT	Alteration	Helman 2018 [23]	Unspecified	Rociletinib	58	1 (2.0) - Co-mutation variants
TP53	Alteration	Helman 2018 [23]	Unspecified	Rociletinib	58	26 (45.0) - Co-mutation variants
TSC1	Alteration	Helman 2018 [23]	Unspecified	Rociletinib	58	1 (2.0) - Co-mutation variants
KRAS, HRAS, NRAS (RAS/RAF signaling pathway)	KRAS Q61H mutation, KRAS K117N mutation, HRAS G12R mutation, and NRAS G10 mutation	Helman 2018 [23]	Unspecified	Rociletinib	65	11 (14.0) - ctDNA positive plasma
PIK3CA, JAK2, BRAF, KRAS, HER2, FGFR3	PIK3CA mutation, JAK2 mutation, BRAF mutation, KRAS mutation, HER2 amplification, FGFR3 TACC3 fusion	Lu 2021 [52]	1L+	Aumolertinib	42	8 (19.0)
RTK or BRAF	Fusion	Schrock 2018 [43]	Unspecified	Erlotinib	12	3 (25.0) - With pre and post treatment NGS samples.
				Afatinib	12	2 (16.7) - With pre and post treatment NGS samples.
				ASP8273	12	1 (8.3) - With pre and post treatment NGS samples.
				Erlotinib then Afatinib	12	1 (8.3) - With pre and post treatment NGS samples.
				Afatinib + Cetuximab	12	1 (8.3) - With pre and post treatment NGS samples.
**EGFR-dependent and EGFR-independent mechanisms**						
EGFR, PIK3CA	EGFR T790M mutation, PIK3CA E542K mutation	Elamin 2022 [51]	1L+	Poziotinib	14	1 (7.1) - S768_D770dupSVD resistance
EGFR, KIT	EGFR T790M mutation, EGFR	Elamin 2022 [51]	1L+	Poziotinib	14	1 (7.1) - D770_N771insG resistance

Gene name	Resistance mechanism	Author, year	LoT	Treatment name	Sample size	N (%) with acquired resistance
	amplification, KIT D910H mutation					
EGFR, MET	EGFR T790M mutation, EGFR amplification, MET amplification	Elamin 2022 [51]	1L+	Poziotinib	14	1 (7.1) - H773_V774VdupHV resistance
EGFR, MET, CDK6	EGFR V774A mutation, MET amplification, CDK6 amplification	Elamin 2022 [51]	1L+	Poziotinib	14	1 (7.1) - H773_V774insAH resistance
EGFR, RET	EGFR T790M mutation, RET NCOA4 fusion	Yao 2019 [47]	Unspecified	Gefitinib	7	1 (14.3) - among cases with RET fusion

Supplementary Table S10. Resistance mutation profile for patients who received unspecified treatment.

Gene name	Resistance mechanism	Author, year	LoT	Sample size	N (%) with acquired resistance
**EGFR-dependent mechanisms**					
EGFR	T790M mutation	Chiang 2020 [19]	1L	47	23 (48.9)
		Gaut 2018 [20]	1L or 2L	97	69 (71.1)
		Chiang 2020 [19]	2L+	25	2 (8.0)
		Li 2019 [28]	Unspecified	23	13 (56.5) - Targeted therapy
		Patil 2020 [35]	Unspecified	7	2 (29.0) - atypical-EGFR mutations
				52	39 (75.0) - typical-EGFR mutations
				34	26 (76.0) - Exon 19del subgroup
				18	13 (72.0) - L858R subgroup
				7	2 (33.0) - G719X subgroup
				2	0 (0.0) - L861Q and exon 20 mutation
		Raez 2022 [38]	Unspecified	3,223	30 (0.9)
	C797S mutation	Raez 2022 [38]	Unspecified	3,223	38 (1.2)
		Li 2019 [28]	Unspecified	23	1 (4.3) - Targeted therapy
	G721S mutation	Raez 2022 [38]	Unspecified	3,223	4 (0.1)

Gene name	Resistance mechanism	Author, year	LoT	Sample size	N (%) with acquired resistance
	G724S mutation	Raez 2022 [38]	Unspecified	3,223	7 (0.2)
	L718V mutation	Raez 2022 [38]	Unspecified	3,223	11 (0.3)
	L747S mutation	Mack 2020 [30]	Unspecified	447	2 (0.4)
	Mutation	Li 2019 [28]	Unspecified	91	13 (14.3)
				13	11 (85.0) - Plasma sample
	T790M mutation, C797S mutation	Li 2019 [28]	Unspecified	NR	2
	T854S mutation	Mack 2020 [30]	Unspecified	447	1 (0.2)
**EGFR-independent mechanisms**					
ALK	Fusion	Mack 2020 [30]	Unspecified	65	11 (16.9)
	F1174C mutation	Mack 2020 [30]	Unspecified	7	1 (14.3) - ≥1 ALK SNV conferring acquired resistance
	F1174V mutation	Mack 2020 [30]	Unspecified	7	1 (14.3) - ≥1 ALK SNV conferring acquired resistance
	G1202R mutation	Mack 2020 [30]	Unspecified	7	4 (41.0) - ≥1 ALK SNV conferring acquired resistance
	I1171T mutation	Mack 2020 [30]	Unspecified	7	2 (28.6) - ≥1 ALK SNV conferring acquired resistance
	L1196M mutation	Mack 2020 [30]	Unspecified	7	2 (28.6) - ≥1 ALK SNV conferring acquired resistance
BRAF	AGAP3 fusion	Schrock 2018 [43]	Unspecified	5	1 (20.0)
	AGK fusion	Schrock 2018 [43]	Unspecified	14	1 (7.1) - With acquired RTK or BRAF Fusions
	ARMC10 fusion	Schrock 2018 [43]	Unspecified	5	1 (20.0)
	GHR fusion	Schrock 2018 [43]	Unspecified	14	1 (7.1) - With acquired RTK or BRAF Fusions
	KIAA1549 fusion	Schrock 2018 [43]	Unspecified	5	1 (20)
	TRIM24 fusion	Schrock 2018 [43]	Unspecified	14	1 (7.1) - With acquired RTK or BRAF Fusions
ERBB2	Amplification	Li 2019 [28]	Unspecified	23	2 (8.7) - Targeted therapy
FGFR3	TACC3 fusion	Schrock 2018 [43]	Unspecified	5	1 (20.0)
HER2	Amplification	Patil 2020 [35]	Unspecified	NR	1
MET	Amplification	Li 2019 [28]	Unspecified	NR	1
		Patil 2020 [35]	Unspecified	9	0 (0) - atypical-EGFR mutations
				31	5 (16) - typical-EGFR mutations
	Mutation	Li 2019 [28]	Unspecified	91	2 (2.2)
RET	RET fusion	Yao 2019 [47]	Unspecified	3600	7 (0.2%)

Gene name	Resistance mechanism	Author, year	LoT	Sample size	N (%) with acquired resistance
	CCDC6 fusion	Yao 2019 [47]	Unspecified	7	5 (71.0) - among cases with RET fusion
	NCOA4 fusion	Yao 2019 [47]	Unspecified	7	2 (29.0) - among cases with RET fusion
	TRIM24 fusion	Schrock 2018 [43]	Unspecified	5	1 (20.0)

Supplementary Table S11 Studies that reported the impact of acquired resistance on clinical outcomes

A. Response

Author year	Patient/treatment groups	Acquired resistance mutation	Sample size	Overall response, %	Complete response, %	Partial response, %	Author reported clinical impact
**Osimertinib**							
Schrock 2018 [43]	Osimertinib (after erlotinib resistance)	EGFR T790M mutation	1	NR	NR	100.0%	The patient received afatinib for 2 months but had mild disease progression. Subsequently, she achieved a PR for 10 months with osimertinib 80 mg once daily.
**Osimertinib with Other Therapies**							
Schrock 2018 [43]	Alectinib + osimertinib (after osimertinib resistance)	EGFR T790M loss, PLEKHA7-ALK fusion	1	NR	NR	100.0%	Alectinib (600 mg twice daily) was added to full-dose osimertinib.<br>The patient had a dramatic and confirmed PR and a duration of response of 6 months.
Le 2022 [16]	Tepotinib + osimertinib or gefitinib (after EGFR-TKI resistance)	MET amplification	12	NR	NR	66.7%	NR
**Other TKIs**							
Helman 2018 [23]	Rociletinib	EGFR T790M mutation – tissue analysis	77	29.9%	NR	NR	NR
		EGFR T790M mutation – plasma analysis	63	28.6%	NR	NR	NR
Yang 2021 [46]	Afatinib (after osimertinib resistance)	EGFR G724S, L718X or C797S mutations	15	36.0%	NR	NR	NR
Bauml 2021-b [55]	Amivantamab + Lazertinib (EGFR exon 19deletion or L858R mutation relapsed on osimertinib)	EGFR/MET mutations or amplifications (biomarker-positive)	17	47.1%	5.9%	41.2%	NR
		Unknown or non-EGFR/MET mechanisms of osi-resistance	28	28.6%	0.0%	28.6%	NR

Author year	Patient/treatment groups	Acquired resistance mutation	Sample size	Overall response, %	Complete response, %	Partial response, %	Author reported clinical impact
		Unknown mechanisms of osi-resistance	18	44.4%	0.0%	44.4%	NR
		Non-EGFR/MET mechanisms of osi-resistance	10	0.0%	0.0%	0.0%	NR
McCoach 2021 [53]	Capmatinib + Erlotinib (after EGFR-TKI resistance)	MET positivity at progression	8	50.0%	12.5%	37.5%	Patient 22, who had a CR, harbored an EGFR L858R mutation and MET amplification and an IHC 3+ score. Patients 26 and 27 both had PRs with EGFR alterations, exon 19 deletion and L858R, respectively, and IHC 3+ but did not demonstrate MET amplification.
**Non-TKIs**							
Janne 2022 [56]	Patritumab Deruxtecan (after EGFR-TKI resistance)	Known EGFR-related resistance mechanisms (excluding T790M)	23	34.8%	NR	NR	In the 23 of 57 patients with known EGFR-related resistance mechanisms (excluding T790M), the confirmed ORR was 35% (CR/PR, 8; SD, 7; PD, 5; NE, 3).
		Known EGFR-independent resistance mechanisms	13	46.2%	NR	NR	In the 13 of 57 patients with known EGFR-independent resistance mechanisms, the confirmed ORR was 46% (CR/PR, 6; SD, 4; PD, 2; NE, 1).
		Other/unknown resistance mechanisms	21	38.1%	NR	NR	In the 21 of 57 patients with other/ unknown resistance mechanisms, the confirmed ORR was 38% (CR/PR, 8; SD, 8; PD, 2; NE, 3).
**Drug/therapy unspecified**							
Gaut 2018 [20]	TKI 1L ± other therapies	EGFR T790M positive	48	75.0%	4.2%	70.8%	NR
		EGFR T790M negative	21	81.0%	0.0%	81.0%	NR
	TKI rechallenge ± other therapies	EGFR T790M positive	18	22.0%	0.0%	22.0%	NR
		EGFR T790M negative	13	0.0%	0.0%	0.0%	NR
	Chemotherapy before or after TKI	EGFR T790M positive	30	40.0%	6.7%	33.3%	NR
		EGFR T790M negative	14	21.4%	0.0%	21.4%	NR
Robichaux 2021 [40]	2nd-generation EGFR inhibitors (e.g., afatinib; after	EGFR pocket volume reducing (PVR) mutation	3	NR	NR	NR	Three patients with NSCLC, who acquired PVR mutations after first-line osimertinib treatment had

Author year	Patient/treatment groups	Acquired resistance mutation	Sample size	Overall response, %	Complete response, %	Partial response, %	Author reported clinical impact
	osimertinib resistance)						clinical benefit after receiving 2nd-generation EGFR inhibitors (e.g., afatinib).

B. **<u>Progression-free survival</u>**

Author year	Patient/treatment groups	Acquired resistance mutation	Sample size	Median (95%CI)	Hazard ratio (95%CI)	Author reported clinical impact
**Osimertinib**						
Cardona 2022 [18]	Osimertinib	RET fusion	4	4.63 months (2.5 - 6.72)	3.13 (1.72 - 4.85)	NR
		RET fusion absent	143	14.68 months (13.2 - 20.2)	Reference	NR
		EGFR T790M loss	23	28.1 months (20.2 - NA)	0.27 (0.13 - 0.55)	NR
		EGFR T790M loss absent	124	13.2 months (11.6 - 16.2)	Reference	NR
		EGFR amplification	12	9.37 months (6.63 - NA)	2.5 (1.28 - 4.7)	NR
		EGFR amplification absent	135	14.4 months (13.1 - 19.8)	Reference	NR
		MET amplification	1	8.57 months (6.72 - 11.3)	21.5 (2.27 - 208.3)	NR
		MET amplification absent	146	14.68 months (13.2 - 20.2)	Reference	NR
		HER2 amplification	9	10.6 months (10.4 - NA)	4.16 (1.85 - 11.1)	NR
		HER2 amplification absent	138	15.1 months (14 - 20.2)	Reference	NR
		PIK3CA mutation	5	11 months (10.7 - NA)	3.57 (1.05 - 13.3)	NR
		PIK3CA mutation absent	142	14 months (12 - 24.6)	Reference	NR
		TP53 mutation	43	NR	NR	While TP53 mutations (considering mutation subtype) were not associated with worse PFS, patients with DNA binding domain mutations in TP53 presented with a worse post-progression survival (PPS)
		TP53 mutation absent	104	NR	NR	

Author year	Patient/treatment groups	Acquired resistance mutation	Sample size	Median (95%CI)	Hazard ratio (95%CI)	Author reported clinical impact
						compared with patients with other types of mutations (1.3 months [95%CI: 1.12-NA] vs. 19.5 months [95%CI: 7.73-NA]; HR: 10.3 [95%CI: 7.82-154.3]; P = 0.002).
	Osimertinib (with CNS metastasis)	TP53 mutation	NR	11.0 months (7.0 - NA)	2.3 (1.1 - 5.0)	Patients who presented with baseline CNS metastases and TP53 mutations at progression had a worse PFS compared with patients with CNS metastases but without TP53 mutations.
		TP53 mutation absent	NR	24.9 months (11.6 - NA)	Reference	
Oxnard 2018 [33]	Osimertinib	EGFR T790M maintained ± EGFR C797S mutation	58	9.5 months (8.2 - 11.0)	P=0.001	Patients with loss of T790M similarly had a shorter PFS.
		EGFR T790M loss	52	4.2 months (3.3 - 6.7)		
**Other TKIs**						
Bauml 2021-b [55]	Amivantamab + Lazertinib (EGFR exon 19deletion or L858R mutation relapsed on osimertinib)	EGFR/MET mutations or amplifications (biomarker-positive)	17	6.7 months (3.4 - NR)	NR	NR
		Unknown or non-EGFR/MET mechanisms of osi-resistance	28	4.1 months (1.4 - 9.5)	NR	NR
Helman 2018 [23]	Rociletinib	EGFR C797S mutation	3	NR	NR	The emerging C797S alteration was first detected 45 to 49 weeks after the initiation of rociletinib treatment and likely drove resistance to rociletinib. These results suggest that the emergence of C797S may result from exposure to third-generation TKIs. Systemic progression occurred after 185 to 358 days of rociletinib treatment.
		KRAS Q61H alteration	1	NR	NR	Patient experienced disease progression on day 118 with the emergence of a new brain lesion. The patient continued to benefit from rociletinib treatment and continued to receive therapy until systemic progression due to a new lesion in the neck occurred on day 297.

Author year	Patient/treatment groups	Acquired resistance mutation	Sample size	Median (95%CI)	Hazard ratio (95%CI)	Author reported clinical impact
		NTRK1 fusion	1	NR	NR	Had stable disease for 168 days while receiving rociletinib treatment until she experienced systemic progression on day 229 due to an increase in target lesions.
		MET amplification	1	NR	NR	Disease progression on day 174 due to the emergence of new brain lesions but continued to receive rociletinib treatment until day 403, when systemic progression due to peritoneal metastasis occurred.
Yao 2019 [47]	Lenvatinib	RET NCOA4 fusion	1	NR	NR	One patient who had EGFR L858R and NCOA4-RET chose lenvatinib, a RET inhibitor, and had a progression free survival of seven months.
**Drug/therapy unspecified**						
Gaut 2018 [20]	TKI 1L ± other therapies	EGFR T790M positive	66	12.0 months (NR)	Reference	Tumors expressing the T790M resistance mutation have a more indolent progression of disease than their T790M negative counterparts as evidenced by a longer PFS on first-line TKI and chemotherapy, though no difference in PFS was noted for TKI rechallenge.
		EGFR T790M negative	28	9.0 months (NR)	Multivariate: 1.75 (1.09 - 2.82) Univariate: 1.46 (0.93 - 2.29)	
	TKI 1L alone	EGFR T790M positive	59	12.0 months (NR)	Reference	
		EGFR T790M negative	26	10.5 months (NR)	1.72 (1.04 - 2.84)	
	TKI 1L ± other therapies that are not chemotherapy	EGFR T790M positive	65	12.0 months (NR)	Reference	
		EGFR T790M negative	28	9.0 months (NR)	1.77 (1.10 - 2.85)	
	TKI rechallenge ± other therapies	EGFR T790M positive	29	4.0 months (NR)	0.97 (0.50 - 1.88)	
		EGFR T790M negative	14	3.0 months (NR)	Reference	
	TKI rechallenge ± other therapies that are not chemotherapy	EGFR T790M positive	23	4.0 months (NR)	0.91 (0.45 - 1.84)	
		EGFR T790M negative	13	3.0 months (NR)	Reference	
	Chemotherapy before or after TKI	EGFR T790M positive	42	5.0 months (NR)	Reference	
		EGFR T790M negative	18	4.0 months (NR)	1.95 (1.09 - 3.49)	

Author year	Patient/treatment groups	Acquired resistance mutation	Sample size	Median (95%CI)	Hazard ratio (95%CI)	Author reported clinical impact
Jin 2019 [26]	EGFR-TKIs	EGFR T790M or RTK-RAS mutations	64	NR	NR	T790M positive patients had improved PFS compared to T790M-negative patients. Baseline and acquired RTK-RAS mutations had opposite effects on PFS.
Robichaux 2021 [40]	2nd generation EGFR inhibitors	EGFR pocket volume reducing (PVR) mutations	NR	24.0 months (NR)	Reference	Retrospective analysis of patients with PVR mutations revealed that patients had a significantly longer median progression free survival when treated with a 2nd-generation inhibitor versus a 1st-generation or 3rd-generation inhibitor.
	1st generation EGFR inhibitors		NR	9.5 months (NR)	4.2 (NR)	
	3rd generation EGFR inhibitors		NR	6.4 months (NR)	3.4 (NR)	

C. Time to treatment discontinuation

Author year	Treatment name	Acquired resistance mutation	Sample size	Median (95%CI)	Author reported clinical impact
**Osimertinib**					
Mondaca 2019 [32]	Osimertinib (2L, after acquired resistance to initial EGFR-TKI)	EGFR T790M detected – lower than median variant allele frequency (VAF)	24	10.3 months (NR)	In 47 patients with ctDNA T790M (+) treated with osimertinib, patients with lower and higher than median VAF had a similar TTD (HR 1.38, 95% CI 0.64–3, p=0.4).
		EGFR T790M detected – higher than median variant allele frequency (VAF)	23	12.7 months (NR)	
Oxnard 2018 [33]	Osimertinib (Institutional cohort)	EGFR T790M maintained	13	15.2 months (range: 11.4 - NA)	Patients with T790M loss had a median TTD of 6.1 months, which was shorter than the median TTD of 15.2 months in patients with maintained T790M (log rank P = .01)
		EGFR T790M loss	28	6.1 months (range: 3.0 - 8.3)	
	Osimertinib (AURA validation cohort)	EGFR T790M maintained ± EGFR C797S mutation	58	12.4 months (range: 10.3 - 14.1)	Patients with loss of T790M had a shorter median TTD (5.5 months) than patients with maintained T790M either without C797S (12.6 months) or with C797S (12.4 months) (log rank P = 0.006).
		EGFR T790M maintained Without EGFR C797S mutation	34	12.6 months (NR)	
		EGFR T790M maintained + EGFR C797S mutation	24	12.4 months (NR)	
		EGFR T790M loss	52	5.5 months (range: 4.3 - 8.3)	
**Osimertinib included as one treatment option among patients treated with TKIs**					
Mondaca 2019 [32]		EGFR T790M detected	56	15.7 months (NR)	

Author year	Treatment name	Acquired resistance mutation	Sample size	Median (95%CI)	Author reported clinical impact
	First-line EGFR-TKI (Erlotinib, Afatinib, Gefitinib, Osimertinib, Rociletinib, or Nazartinib)	EGFR T790M not detected	121	16.9 months (NR)	The TTD of first-line EGFR-TKI in ctDNA T790M (+) and (–) patients was 15.7 months and 16.9 months, respectively (HR 1.32, 95% CI 0.93–1.88, p=0.09).

D. Overall survival

Author year	Patient/treatment groups	Acquired resistance mutation	Sample size	Median (95%CI)	Hazard ratio (95%CI)	Survival at 3 months n (%)	Survival at 6 months n (%)	Survival at 12 months n (%)	Author reported clinical impact
**Osimertinib**									
Mondaca 2019 [32]	Osimertinib (2L, after acquired resistance to initial EGFR-TKI)	EGFR T790M detected – lower than median variant allele frequency (VAF)	24	16.6 months (NR)	1.17 (0.42 - 3.2)	19 (91.6)	14 (77.8)	6 (65.2)	In 47 patients with ctDNA T790M (+) treated with osimertinib, patients with lower and higher than median VAF had a similar OS (HR 1.17, 95% CI 0.42–3.2, p = 0.76)
		EGFR T790M detected – higher than median variant allele frequency (VAF)	23	Not reached (NR)	Reference	20 (91.0)	15 (82.0)	8 (75.6)	
**Osimertinib included as one treatment option among patients treated with TKIs**									
Mondaca 2019 [32]	First-line EGFR-TKI (Erlotinib, Afatinib, Gefitinib, Osimertinib, Rociletinib, or Nazartinib)	EGFR T790M detected	56	4.6 years (NR)	1.39 (0.77 - 2.48)	NR (100.0)	NR (98.2)	51 (94.9)	There was no difference in OS between these two groups (median OS 4.6 vs. 7.6 years, HR 1.39, 95% CI 0.77–2.48, p = 0.24).
		EGFR T790M not detected	121	7.6 years (NR)	Reference	NR (100.0)	NR (98.0)	107 (94.1)	
